# Integrated Multi-omics Reveals Molecular Signatures of Rett Syndrome, Informing Future Biomarker Discovery

**DOI:** 10.1007/s12035-026-06296-8

**Published:** 2026-11-09

**Authors:** Jasmeen Kaur Issar, Ashley Hertzog, Ignatius Pang, Mark E. Graham, Nader Aryamanesh, Lisa G. Riley, Ignazio S. Piras, Nathaniel P. Hansen, Caitlin A. Finney, Artur Shvetcov, Brian Gloss, Sushil Bandodkar, Russell C. Dale, Qin Sun, Carolyn Ellaway, Adviye A. Tolun, Gladys Ho, Walter E. Kaufmann, Wendy A. Gold

**Affiliations:** 1 School of Medical Sciences (Neuroscience), The Faculty of Medicine and Health, University of Sydney, Sydney, NSW Australia; 2 Kids Neuroscience Centre, Kids Research, The Children’s Hospital at Westmead, Westmead, NSW Australia; 3 NSW Biochemical Genetics Service, The Children’s Hospital at Westmead, Westmead, NSW Australia; 4 Australian Proteome Analysis Facility, Macquarie University, Sydney, NSW Australia; 5 Biomedical Proteomics, Children’s Medical Research Institute, The University of Sydney, Sydney, NSW Australia; 6 Bioinformatics Group, Children’s Medical Research Institute, Faculty of Medicine and Health, University of Sydney, Sydney, NSW Australia; 7 Early Detection and Prevention Division, Translational Genomics Research Institute, Phoenix, AZ USA; 8 Translational Dementia Research Group, Westmead Institute for Medical Research, Sydney, Australia; 9 Department of Psychological Medicine, Sydney Children’s Hospitals Network, Sydney, NSW Australia; 10 Discipline of Psychiatry and Mental Health, School of Clinical Medicine, Faculty of Medicine and Health, University of New South Wales, Sydney, NSW Australia; 11 Westmead Research Hub, Westmead Institute for Medical Research, Sydney, NSW Australia; 12 Department of Biochemistry, The Children’s Hospital at Westmead, Westmead, NSW Australia; 13 The Children’s Hospital at Westmead Clinical School, Faculty of Medicine and Health, University of Sydney, Sydney, NSW Australia; 14 Department of Molecular & Human Genetics, Baylor College of Medicine, Houston, USA; 15 Baylor Genetics, Houston, USA; 16 Genetic Metabolic Disorders Service, Sydney Children’s Hospitals Network, Sydney, NSW Australia; 17 Department of Molecular Genetics, The Children’s Hospital at Westmead, Westmead, NSW Australia; 18 Department of Human Genetics, Emory University School of Medicine, Atlanta, GA USA; 19 Department of Neurology, Boston Children’s Hospital, Boston, MA USA

**Keywords:** Rett syndrome, Blood, Biomarker, Omics, MeCP2, Neurodevelopment

## Abstract

Rett syndrome (RTT) is a severe neurodevelopmental disorder primarily caused by pathogenic variants in the X-linked *methyl-CpG-binding protein 2* (*MECP2*) gene. Despite numerous clinical trials being conducted, there are limited disease-modifying options available for individuals with RTT. This paucity of effective treatments can in part be attributed to the lack of clinically useful biomarkers that can objectively assess treatment response. Moreover, diagnosis of RTT continues to be clinical, when clinical manifestations are apparent, precluding early therapeutic approaches. Thus, clinical biomarkers of disease stage could improve diagnosis and enable real-time monitoring of response to treatment, providing the earliest possible indications of therapeutic efficacy. Given MeCP2’s role as a key regulator of gene transcription, we used an untargeted omics approach to evaluate transcriptomic, proteomic, and metabolic profiles in blood from individuals with RTT (*n* = 9) and age- and sex-matched controls (*n* = 8). In addition to characterising differential expression at each omic layer, to understand the shared biology and coordinated molecular signals across all omic datasets, we used the unsupervised machine learning framework Multi-Omics Factor Analysis version 2 (MOFA2). The individual omic data consisted of a robust upregulation of genes involved in ribosomal structure and translation together with a downregulation in vesicle trafficking genes. An upregulation in ribosomal machinery and mitochondrial function together with a concomitant downregulation of critical epigenetic regulators, immune-signalling molecules, and cytoskeletal structural anchors was observed in the proteomics, which was supported by the phosphoproteomics. Notably, plasma metabolomics pointed towards alterations in energy substrates and lipid/cholesterol handling, suggesting systemic downstream consequences of primary intracellular disturbances. The integrated MOFA2 data supported the individual omic data revealing a central disease axis principally shaped by the proteome and transcriptome and supported by the metabolome and phosphoproteome, identifying potential biomarkers, including latent biological processes that distinguish RTT from controls (Latent Factor 1) and correlates of clinical severity (Latent Factor 2). Overall, our data reveal a distinctive ‘disease state’ molecular signature that distinguishes individuals with RTT from controls, where the findings in RTT blood cells are consistent with previously reported altered translational and mitochondrial function and disrupted chromatin regulation, cytoskeletal dynamics, and metabolic signalling. Our integrated multi-omics analyses revealed that PBMC’s LTN1, ATRX, CHD8, SPTB, DMTN, PRF1, CD40LG, and PTPRCAP and plasma glutamate are the most promising disease-state (complementary diagnostic) candidate biomarkers while sphingomyelin, RTN3, and MMP1 appear to be the most suitable biomarkers of disease severity. Here, we demonstrate that blood from individuals with RTT reflects the molecular systemic consequences of MeCP2 dysfunction.

**Supplementary Information:**

The online version contains supplementary material available at https://doi.org/10.1007/s12035-026-06296-8.

## Introduction

Rett syndrome (RTT) is a severe X-linked neurodevelopmental disorder that affects approximately 1 in 10,000 female births and is caused predominantly by pathogenic de novo loss of function variants in the X-linked gene *MECP2* that encodes methyl-CpG-binding protein 2 [1, 2]. As a master regulator of chromatin organisation and gene expression, MeCP2 influences multiple biological processes, including neuronal maturation, synaptic plasticity, metabolism, and immune function [2, 3]. Loss of MeCP2 function results in multiple neurological abnormalities and a complex evolution that includes developmental delay, a distinctive developmental regression, and progressive impairments. Core and common clinical manifestations include partial or complete loss of acquired spoken language and hand function, hand stereotypies, gait disturbances, intellectual disability, epilepsy, behavioural issues, autonomic dysfunction, breathing abnormalities, and a range of multisystem comorbidities such as constipation, decreased bone density, and scoliosis.

While treatment of RTT remains symptomatic, the therapeutic landscape for RTT has changed substantially over the past decade. The FDA approval of trofinetide (DAYBUE®), a synthetic version of a tripeptide derived from IGF-1, which targets core symptoms of the disorder, together with the rapid development of genetic therapies either in clinical trials or moving towards them, has shifted the field from symptomatic management towards disease modification [4]. Consequently, there is an increasing need for objective biomarkers capable of supporting patient subtyping, monitoring early ‘real-time’ response to therapy, and providing mechanistically relevant outcomes for clinical trials. Furthermore, recent pilot newborn screening data on *MECP2* suggest that even presumably pathogenic variants are not predictive of a RTT phenotype [5] raising concerns about early diagnosis and implementation of novel therapies. Although promising neurophysiologic biomarkers are under development [6], there are no objective molecular biomarkers that provide insights into RTT’s underlying pathophysiology and can be used as surrogate endpoints in treatment studies. The RTT field still relies on clinician- and caregiver-reported outcome measures, such as the Rett Syndrome Behaviour Questionnaire (RSBQ) and the Clinical Global Impression - Improvement (CGI-I), which are not well integrated and are not particularly suited for evaluating changes in RTT’s evolution as those expected from disease-modifying therapies [7, 8]. The need for early diagnosis in order to administer treatments that can prevent the progression of RTT, in the context of disappointing initial results of newborn screening studies [5], has highlighted the need for identifying biomarkers that are distinctive and, hopefully, also specific of the disorder.

Blood represents an attractive source for biomarker discovery because it is readily accessible, minimally invasive, and amenable to repeated sampling throughout the course of clinical trials. Although peripheral tissues cannot directly recapitulate neural dysfunction, numerous studies have demonstrated that alterations in immune signalling, mitochondrial function, cellular metabolism, and protein homeostasis observed in the brain are also reflected in circulating blood cells and plasma [9]. Blood-based biomarkers, therefore, have the potential to provide real-time information regarding systemic consequences of MeCP2 dysfunction while offering a practical platform for longitudinal monitoring in both clinical practice and therapeutic studies.

Historically, many studies have utilised targeted methodologies in the pursuit of identifying biomarkers for RTT. These studies have shown dysregulation in biogenic amine metabolites and oxidative markers in affected individuals and animal models [10–12]. Additionally, studies performing individual untargeted genomics, proteomics, and metabolomics analyses in cohorts of patients (and animal models) with RTT have also been successful in identifying biomarkers and dysregulated pathways [13–18]. While there are some reports of untargeted omics studies being performed in tissue samples obtained by minimally invasive methods [14, 19], it is important to note that a significant portion of these types of studies have used tissue types collected by invasive measures (i.e. lumbar puncture and skin biopsy) [13, 15, 20] or postmortem [21]. Routine and frequent analyses of invasive biosamples are not pragmatic in a clinical care setting, nor in clinical trials.

Recent advances in high-throughput omics technologies now permit the simultaneous interrogation of the transcriptome, proteome, phosphoproteome, and metabolome from the same individuals [22]. Each omics modality provides complementary biological information, capturing distinct levels of gene regulation, protein abundance, post-translational modification, and metabolic activity. However, analysing each dataset independently often fails to identify the coordinated biological programmes driving disease because important relationships between molecular layers remain obscured. Multi-Omics Factor Analysis version 2 (MOFA2) is an unsupervised machine learning framework specifically developed to integrate heterogeneous multi-omics datasets [22]. Rather than analysing each molecular layer independently, MOFA2 identifies latent factors that capture coordinated sources of biological variation shared across datasets. These latent factors provide biologically meaningful representations of disease-associated molecular programmes while simultaneously identifying the molecules contributing most strongly to each source of variation. Such approaches offer considerable advantages over traditional differential expression analyses by revealing systems-level mechanisms that may underpin disease pathogenesis, patient heterogeneity, and clinical severity.

Given the role of *MECP2* and its protein’s broad impact on CNS and peripheral physiology, a multi-dimensional approach that interrogates the ‘whole of organism’ phenotype is likely needed. Here, we performed an integrated multi-omics analysis of peripheral blood collected from individuals with classic RTT and age-matched controls. Transcriptomic, proteomic, phosphoproteomic, and metabolomic datasets were analysed independently before integrating them using MOFA2 to identify coordinated molecular programmes and latent factors associated with the disorder. We further investigated whether latent molecular factors also correlated with clinical severity and validated a selected candidate biomarker using orthogonal biochemical approaches. We hypothesise that integrated blood multi-omics can identify distinct molecular signatures capable of both distinguishing RTT from healthy controls and quantifying disease severity, thereby providing objective biomarkers to support diagnosis and management of the disorder.

## Methods

### Participants

Females with a clinical diagnosis of classical RTT [23] carrying pathogenic variants in *MECP2* were recruited to participate in the study through the multidisciplinary RTT clinic at the Children’s Hospital at Westmead (Westmead, NSW, Australia) (Table 1). Age- and sex-matched controls with no known significant health conditions (including neurodevelopmental disorders) were also recruited. Peripheral blood samples were collected from nine individuals with pathogenic variants in *MECP2* (median age of 12.4 years, range 4.1–18.3 years) and eight age- and sex-matched controls (median age of 15.3 years, range 9–19.4 years). For each participant, venous blood samples were collected during a single visit into an ACD vacutainer tube and an EDTA vacutainer tube. Peripheral blood mononuclear cells (PBMCs) were isolated from the ACD tube by Ficoll-Paque density-gradient centrifugation and divided into aliquots for transcriptomic and proteomic/phosphoproteomic analysis from the same cell pool for each individual; the plasma in the EDTA tube was centrifuged and the supernatant was aliquoted for metabolomic profiling. Genotype, demographics, and clinical severity, based on the Clinical Severity Scale [24] and medication information, of the RTT cohort are summarised in Table 1. At the time of blood collection, all study participants were stable and there were no reported significant medical events (including infections) in the preceding 2 weeks. Written and informed consent was taken. The study was approved by the Sydney Children’s Hospital Network ethics committee (13/SCHN/14). Information on genotype, clinical severity, and current medication use was obtained through the hospital electronic medical record.

**Table 1:** Genotype, demographics, clinical severity, and medication information in individuals with Rett syndrome and controls

ID	Participant	Genotype (NM_004992.4)	Clinical severity score [24]	Age at collection (years)	Medications
C1	Unaffected	N/A	N/A	15.0	NA
C2	Unaffected	N/A	N/A	15.6	NA
C3	Unaffected	N/A	N/A	12.8	NA
C4	Unaffected	N/A	N/A	12.7	NA
C5	Unaffected	N/A	N/A	9.0	NA
C6	Unaffected	N/A	N/A	15.5	NA
C7	Unaffected	N/A	N/A	19.2	NA
C8	Unaffected	N/A	N/A	19.4	NA
P1	RTT	c.378-3C > G p.?	38	18.2	Lamotrigine, macrogol, sustagen*
P2	RTT	c.763C > T p.(Arg255*)	25	18.3	Carbamazepine, levetiracetam, vitamin D, benefibre (wheat dextrin), armaforce supplement**, sustagen*
P3	RTT	c.502C > T p.(Arg168*)	10	18.3	Levetiracetam, fluoxetine, risperidone, iron, vitamin D, calcium, vitamin C, fish oil
P4	RTT	c.808delC p.(Arg270Glufs*19)	21	12.4	Levetiracetam, lamotrigine, macrogol
P5	RTT	c.1107_1196delinsA p.(His369Glnfs*6)	34	13.6	Perampanel, lamotrigine, sodium valproate, topiramate, zonisamide, levetiracetam, esomeprazole, macrogol, melatonin, oral contraceptive pill
P6	RTT	c.917 G > A p.(Arg306His)	30	9.0	Cannabidivarin, macrogol, potassium citrate, cephalexin prophylaxis
P7	RTT	c.473C > T p.(Thr158Met)	9	7.4	Probiotic
P8	RTT	c.808C > T p.(Arg270*)	20	4.1	None
P9	RTT	c.880C > T p.(Arg294*)	16	6.6	Sodium valproate, vitamin D, magnesium, fish oil

*Mixture of milk solids, sugar/maltodextrin, whey protein, vitamins A, B complex, C, D, E, K) and several minerals including calcium

**Immune system supplement

### RNA Sequencing and Bioinformatics for Individual Analysis

PBMCs that were isolated as described above were used for RNA extraction. Illumina sequencing was performed, and the RNA-Seq raw fastq files were quality-checked with *FastQC*, and adapter trimming and quality filtering were performed with *AdapterRemoval*. Trimmed and quality-filtered paired-end reads were aligned to the human GRCh38 reference genome using *STAR* with default parameters [25]. Finally, gene expression was quantified by counting reads aligned to each Ensembl gene model using *STAR*. Differentially expressed gene (DEG) analysis was performed using the *Limma-Voom* method [26, 27]. The trimmed mean of M-values (TMM) normalisation method from *edgeR* was applied to normalise read counts according to library size differences between samples [28]. Genes with < 2 count per million (CPM) reads in a minimum of three samples were removed. After filtering and normalisation, removal of unwanted variation was conducted using the remove unwanted variation (RUV) package [29]. A factor of unwanted variation (*k* = 4) was applied to adjust for technical noise and eliminate genes exhibiting minimal differential expression relative to the designated negative control genes. Genes are considered differentially expressed if their false discovery rate (FDR) adjusted *p*-value was < 0.05. DEGs were visualised using a volcano plot using the R package *ggplot2*, and heatmaps were created using the *dittoSeq* package.

### Proteomic and Phosphoproteomic Sample Preparation

PBMCs isolated as described above were lysed to generate whole-cell lysates for proteomic and phosphoproteomic analyses. Tandem mass tag (TMT) labelling was performed with TMTpro reagents using approximately 200 μg of tryptic peptides per sample, and the combined TMT-labelled samples were desalted using solid-phase extraction. Phosphopeptide enrichment was performed as previously described [30], and the eluate was subjected to hydrophilic interaction liquid chromatography separation as previously described [31]. LC-MS/MS analysis was performed using a Dionex UltiMate 3000 RSLC nano system and Q Exactive Plus hybrid quadrupole-orbitrap mass spectrometer.

### LC-MS/MS Phosphopeptide-Enriched Fractions

Samples were loaded onto the column in 99% reversed phase buffer A (solution of 0.1% formic acid) and 1% buffer B (solution of 0.1% formic acid, 90% acetonitrile (34,967, Honeywell)). Data-dependent LC-MS/MS acquisition was performed at a resolution of 70,000 with an automatic gain control target of 1,000,000 for a maximum ion time of 100 ms from m/z 375 to 1500. The MS/MS scans were at a resolution of 35,000 with an automatic gain control target of 200,000 and a maximum ion time of 115 ms. The loop count was 12, the isolation window was 1.1 units of the m/z scale, the first mass was fixed at m/z 120, and the normalised collision energy was 34. Singly charged ions and those with charge > 8 were excluded from MS/MS and dynamic exclusion was for 35 s.

### LC-MS/MS Non-phosphopeptide-Enriched Fractions

Samples were loaded onto the column in 1% buffer B. Data-dependent LC-MS/MS acquisition was performed for 140 min. Scan settings were as for phosphopeptides, except that the normalised collision energy was 31 and dynamic exclusion was set to 37 s.

### Differential Abundance Protein Analysis and Linear Modelling

The proteomics analysis and linear modelling were performed as previously described unless otherwise stated below [32]. A set of 500 empirical negative control proteins with little or no change in protein expression across samples (*q*-value > 0.05) was identified from an initial ANOVA test. Significant differentially expressed proteins were defined as those with *q*-values < 0.05. Differentially expressed proteins were analysed for pathway enrichment in Reactome pathways and Gene Ontology (GO) terms using the clusterProfiler R library [33]. Redundant GO terms were removed using REVIGO [34] using the medium redundancy setting of 0.7.

### Differential Abundance Phosphopeptide Analysis and Linear Modelling

To generate ‘evidence.txt’ files, the raw LC-MS/MS data was processed with MaxQuant v1.6.7.0. Any phosphopeptides were removed from further analysis if the first entry returned by MaxQuant matched any entries in the contaminants or the reverse sequence decoy databases (where the protein accession was prefixed with CON_ or REV_, respectively). Phosphopeptides with any missing values were removed from the analysis. If the phosphopeptide mapped to multiple proteins, the ‘best’ protein annotation was determined for each phosphopeptide, and this was recorded as a multi-mapped phosphopeptide. The best UniProt accessions and the best gene symbol for the multi-mapped proteins were kept for further analysis. The multiple UniProt accessions and multiple gene symbols were separated by a colon ‘:’ and were filtered for high-confidence phosphopeptides and a summary of abundance values.

Identification of phosphopeptides that were of high confidence involved the following two steps. Firstly, to identify all phosphopeptides in which every major phosphorylation site had a site probability score of ≥ 0.75 (including analysing the site probability scores for all major sites for multiply phosphorylated peptides). All phosphorylation sites identified were called high confidence phosphorylation sites. Secondly, to identify all phosphopeptides in which every major phosphorylation site had a site probability score of > 0.5, and the site has also been previously identified as a high confidence phosphorylation site. The abundance values of all phosphopeptides identified using the above criteria were kept for further differential abundance analysis. The abundance values from different phosphopeptides sharing the same group of phosphorylation sites were summed. This resulted in the total abundance values corresponding to each group of phosphosites shared by multiple phosphopeptides. For simplicity, these groups of co-occurring phosphorylation sites are referred to as phosphosites throughout the “Results” section and figure legends, where phosphosites are mapped to their parent protein for downstream Reactome/GO pathway enrichment analyses, the corresponding entities are referred to as phosphoproteins.

Data normalisation was similar to the method utilised for the proteomics analysis described above. Between sample normalisation was performed using the ‘cyclicloess’ normalisation from the ‘limma’ R package. The RUVIII package was used to remove batch effects, and the parameters used included selecting 1000 phosphopeptides with the least changes across samples as negative control proteins and four unwanted components (*k* = 4) were removed by the tool. For host protein normalisation of the phosphopeptide, the host protein’s log fold-change was subtracted from the log fold-change of the phosphopeptide. The host protein normalised phosphopeptide log fold-change was used for all subsequent analysis. If the direction of the fold-change for the phosphopeptide and the direction of the fold-change of the protein were opposite, the *q*-value of the phosphopeptide and the *q*-value of the protein were used directly for normalisation using Fisher’s method (resulting in a more statistically significant *q*-score for the phosphopeptide). If the direction of the log-fold change between the phosphopeptides and proteins were the same, then 1 minus the protein *q*-value and the original phosphopeptide *q*-value were used for normalisation using the Fisher’s method (resulting in a less significant *q*-score for the phosphopeptide).

Differential abundance analysis of phosphopeptides was performed to compare individuals with RTT to controls. The statistical analysis was similar to that of the quantitative proteomics analysis above, in which the ‘limma’ R package [27], linear modelling, and empirical Bayes methods were used to calculate the moderated *p*-values for the comparison of individuals with RTT to controls. A FDR correction was applied using *q*-values [35]. Significantly differentially expressed phosphopeptides were defined as those with *q*-values < 0.05. Proteins containing significant phosphosites were separated into three subsets for pathway enrichment with those containing only significantly increased phosphosites, only significantly decreased phosphosites, or a mixture of both, and each subset was tested for enrichment against Reactome and GO terms as described above for the proteomics analysis.

The ‘KinSwingR’ R library [31] was used to perform kinase-substrate enrichment to infer upstream kinases with up- or downregulated activities in the experiment. To de-duplicate multi-phosphorylated peptides containing common phosphorylation sites, for each phosphorylation site present in common across multi-phosphorylated peptides, the greatest log fold-change of the phosphosite across all phosphopeptides and the corresponding *q*-value were kept for KinSwingR analysis. Known phosphosites annotated with kinase-substrate interactions from all mammalian species (from PhosphositePlus version DB5) were used to train kinase-specific scoring matrices. The KinSwingR analysis was then proceeded with default parameters as previously described [31].

### Metabolomic Analysis and Linear Modelling

Plasma isolated as described above was aliquoted for shipment to Baylor Genetics (Houston, TX, USA), where clinical untargeted metabolic profiling was performed by Metabolon Inc., Morrisville, NC, USA and Baylor Genetics as previously described [36, 37].

Metabolomics analysis was performed using a method similar to the proteomics analysis using RUVIII-C and limma, unless stated below. A set of 150 empirical negative control metabolites with little or no change in metabolite abundance across samples (*q*-value > 0.05) was identified from an initial ANOVA test. The RUVIII method [38] was used to remove unwanted variation across samples, and the tool removed six unwanted components (*k* = 6). Next, the missForest R package [39] was used to impute missing values. The parameters for missForest used were maximum number of iterations (maxiter) = 10 and random number seed was 12,346. Parallelization of computation was performed using the ‘variables’ method. Significantly differentially expressed metabolites were defined as those with *q*-values < 0.05. Pathway enrichment analysis was performed using the ‘camera’ function in the limma R package and the metabolomic pathways from the KEGG database.

### Integrative Multi-omics Factor Analysis v2(MOFA2)

#### Transcriptomics

For integration, a set of 200 empirical negative control transcripts, with little or no change in protein expression across samples (*q*-value > 0.05), was identified from an initial ANOVA test. The RUVIII method [38] was used to remove the unwanted variations across the samples and a number of unwanted components (*k* = 6) were removed prior to input into the multi-omics analysis tool MOFA2.

#### Proteomics and Phosphoproteomics

The RUVIII normalised phosphosite intensity for each protein in each sample was divided by the RUVIII normalised protein intensity for that protein in that sample. This resulted in a matrix of phosphosite intensities, which had been adjusted for host protein abundance. This matrix was used for input into the integrative multi-omics analysis tool MOFA2.

#### Metabolomics

The RUVIII normalised metabolite intensity values were used for input into the integrative multi-omics analysis tool MOFA2.

#### MOFA2

Multi-omics data was merged using the unique patient and control sample identifier as joining criteria. Analysis was performed with transcriptomic, proteomic, phosphoproteomic (adjusted for host protein abundance), and metabolomic data. MOFA2 was run as applied in the R package MOFA2 [22] on the merged data using the basilisk option set to ‘TRUE’ to ensure reproducible dependency management. All other default (gaussian) parameters were maintained. The input into MOFA2 was four data modalities including genes, protein, phosphosites, and metabolites data after preliminary filtering and RUVIII normalisation described above. The primary analyses intended to identify latent factor(s) discriminating patients with RTT from controls. To investigate the relationship between patients’ clinical severity and the latent factors identified by MOFA2, Spearman’s rank correlation was calculated between the Clinical Severity Scale (CSS) and the sample weights for each factor. To identify the specific molecules driving this association, the top 10 features (transcripts, proteins, phosphosites, and metabolites) with the highest loading scores on Factor 2 were identified.

#### MOFA2: Correlation Network

The functional relationships among the molecules were established by identifying the top 15 molecules from each omics layer based on the absolute weights in Factor 1, the one providing the best discrimination between RTT and control samples, from the MOFA2 analysis. The RUVIII normalised log base-2 quantities for each of these molecules were Z-transformed (mean-centred and scaled to unit variance) such that each molecule had a mean of zero and a standard deviation of one across all samples. For the RTT samples, the Pearson correlation score and associated *p*-value for the correlation score for each pair of molecules were calculated and the same was done separately for the control samples. For each pair of molecules, the differential correlations between the individuals with RTT and the controls (RTT Pearson correlation − the control Pearson correlation) were calculated. Only pairs of molecules that had differential correlation of < − 1 or > 1 (and *p*-value < 0.05 for either the RTT correlation or the control correlation) were included in the network.

#### Orthogonal Validation Assays

Orthogonal validation was performed for the most informative plasma metabolite, in this case glutamate, because of its relevance as circulating systemic (overall) signature of MeCP2 dysfunction. For this purpose, plasma from RTT individuals and controls was analysed in the New South Wales Biochemical Genetics Service’s diagnostic laboratory. Samples were deproteinised in a 1:1 volume of a 5-sulphosalicylic acid-containing internal standard mixture and centrifuged at 18,000 rpm at 4 °C for 5 min. A 1:1:3 ratio of the protein-free supernatant to 6-aminoquinolyl-N-hydroxysuccinimidyl carbamate derivatising reagents to NaOH/Borate buffer was added into an HPLC total recovery vial, vortexed, and left at room temperature for 60 s. The mixture was then incubated at 55 °C for 10 min. Chromatographic separation occurred using a reverse phase BEH C18 column and a water/acetonitrile solvent gradient by ultra-performance liquid chromatography coupled to an ultraviolet light detector (UPLC-UV). GraphPad Prism 9.5.1 was used for all statistical analyses, with a significance threshold of *p* < 0.05. Results are expressed as medians and are displayed with graphs depicting the interquartile ranges. The Mann-Whitney *U* test was used to compare independent continuous variables. The relationship between plasma glutamate concentration and CSS was assessed using Spearman rank correlation and the coefficient of determination (*R*^2^) from an ordinary least-squares linear regression (*R*^2^ = 0.009).

## Results

### Study Participants

**Fig. 1:**
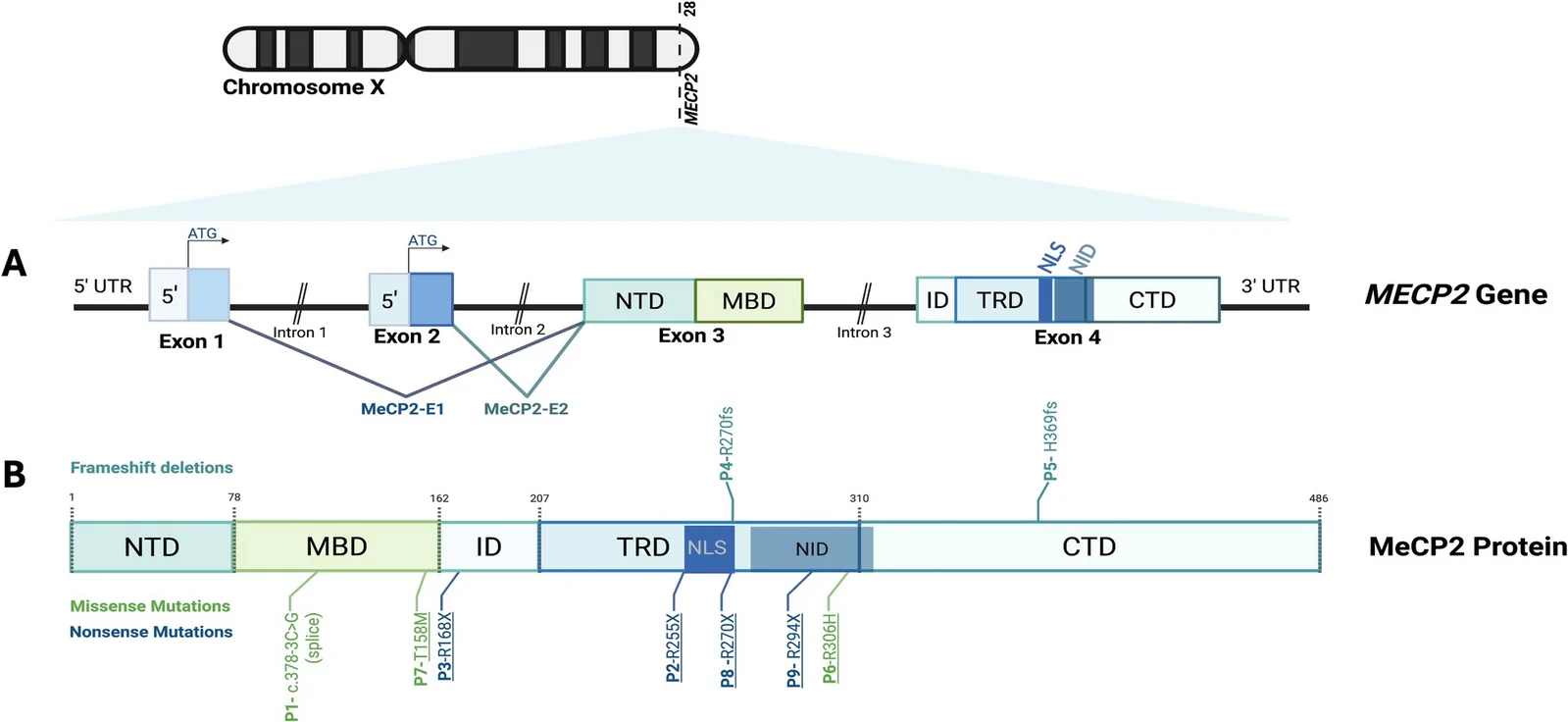
*MECP2* gene, MeCP2 protein, and common variants in Rett syndrome. **A** Structure of the *MECP2* gene MeCP2 protein domains. The two mRNA isoforms, *MECP2E1* and *MECP2E2*, code for two protein isoforms, which differ in their amino termini due to alternative translation start sites and selective inclusion of exons 1 or 2 in the *MECP2* transcript. **B** Structure of the MeCP2 protein and location of variants in this cohort. NTD, N-terminal domain; MBD, methyl-CpG-binding domain; ID, interdomain; TRD, transcriptional repression domain; NLS, nuclear localisation signal; NID, NCoR/SMRT interaction domain; CTD, C-terminal domain

The *MECP2* variants, clinical severity scores (CSS) [24], and medications of the RTT cohort (*n* = 9) are summarised in Table 1 and Fig. 1. All affected individuals were diagnosed with classic RTT, carried a variety of *MECP2* variants with 56% being hot spot mutations (T158M, R168X, R255X, R270X, and R294X) (Fig. 1), had a mean overall CSS score of 22.56 (range 9–38), and had a median age of 12.4 years (range 4.1–18.3 years). The sex-matched controls had a median age of 15.3 years (range 9–19.4 years).

### Blood Multi-omics Consistently Separates Rett Syndrome from Control Groups Across Transcriptomics, Proteomics, Phosphoproteomics, and Metabolomics Using PCA

To determine whether peripheral blood of individuals with RTT captured a molecular signature across omics layers, we first examined each omics dataset independently using principal component analysis, allowing us to assess whether transcriptomic, proteomic, phosphoproteomic, and metabolomic profiles could distinguish individuals with RTT from age- and sex-matched controls.

**Fig. 2:**
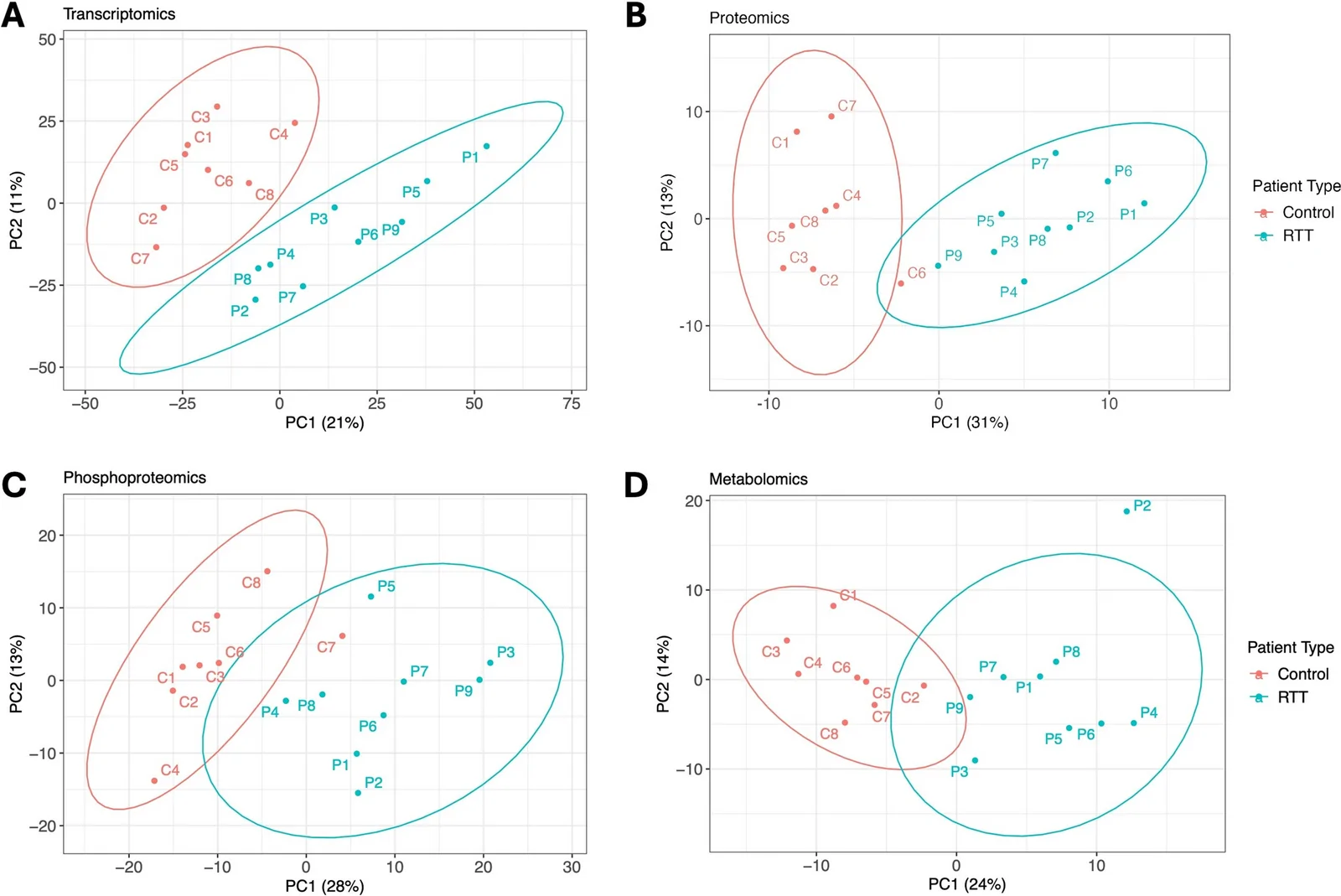
Principal component analysis (PCA). **A** Transcriptomics data. **B** Proteomics data. **C** Phosphoproteomics data. **D** Metabolomic data. The four sets of plots show distinct clustering of individuals with RTT (blue) versus controls (red)

While the transcriptomic alterations were subtle, they demonstrated biologically meaningful and internally consistent disease-associated signatures. The transcriptomics principal component analysis (PCA) showed a distinct separation between individuals with RTT and controls across the first (21%) and second (11%) principal components (Fig. 2A). The proteomic layer provided a far stronger and more disease-discriminating signal than the transcriptome, where the PCA showed a clear separation between individuals with RTT and controls across the first (31%) and second (13%) principal components (Fig. 2B). Phosphoproteomics captures the dynamic state of intracellular signalling and structural regulation. The PCA separation across the first (28%) and second (13%) phosphoproteomics principal components was robust, though slightly less distinct than that of total proteomics (Fig. 2C). Plasma metabolomics captures the systemic milieu, and it showed clean RTT-control discrimination across the first (24%) and second (14%) principal components (Fig. 2D). Consistent separation across every molecular layer confirms that RTT biology is highly coordinated, thus warranting an integrative approach.

### Differential Expression Suggests Coordinated Dysregulation Across Omic Layers Including Ribosomal, Mitochondrial, Chromatin, Cytoskeletal, Immune, and Metabolic Pathways

**Fig. 3:**
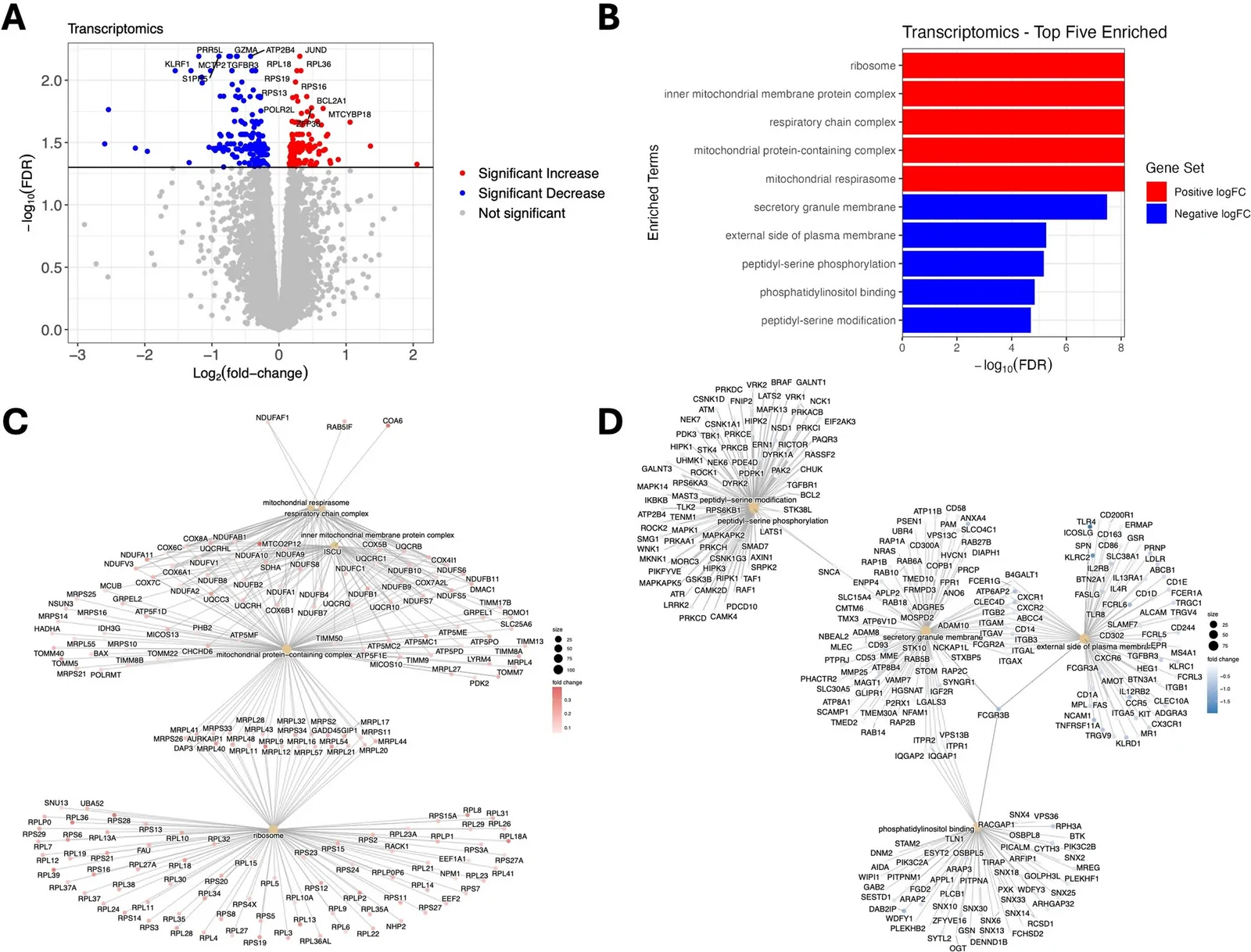
Transcriptomics data. **A** Volcano plot depicting differentially expressed genes showing fold-changes (*x*-axis) and statistical significance (−log10FDR, *y*-axis) with increased abundance in red and decreased abundance in blue (FDR < 0.05). **B** Bar chart showing top 5 enriched Gene Ontology (GO) pathways with increased (red) and decreased gene expression (blue). **C** Gene-concept network (CNET) plot linking genes most significantly increased in expression from GO pathways. **D** CNET plot linking genes most significantly decreased in expression from GO pathways

A relatively low number of differentially expressed genes (DEGs) with modest fold-changes (< log2FC for most) were identified (Fig. 3A). A total of 350 genes were differentially expressed; 149 were significantly upregulated (FDR *p*-value < 0.05), enriched in genes that encode for ribosomal proteins (*RPL18*, *RPL36*, *RPS13*, *RPS16*, and *RPS19*) and protein translation components, and 201 were significantly downregulated (FDR *p*-value < 0.05), including genes encoding immune signalling and secretory function proteins. Pathway enrichment analysis utilising Gene Ontology (GO) identified that genes with increased expression in the RTT cohort were enriched in pathways related to ribosomal structure and mitochondrial energy production, while genes with decreased expression were enriched in pathways related to secretory granule membrane components essential for regulating secretion (Fig. 3B). The gene-concept network (CNET) plot, which links enriched pathway terms with genes annotated with the specific pathway term, is shown for GO pathways (Fig. 3C and D). Genes encoding mitochondrial ribosomal subunits (*MRPL*) and cytoplasmic ribosomal subunits (*RPS* and *RPL*) were increased in expression in individuals with RTT (Fig. 3C), and genes encoding proteins involved in secretory granule membrane function and plasma membrane communication were decreased in expression (Fig. 3D).

**Fig. 4:**
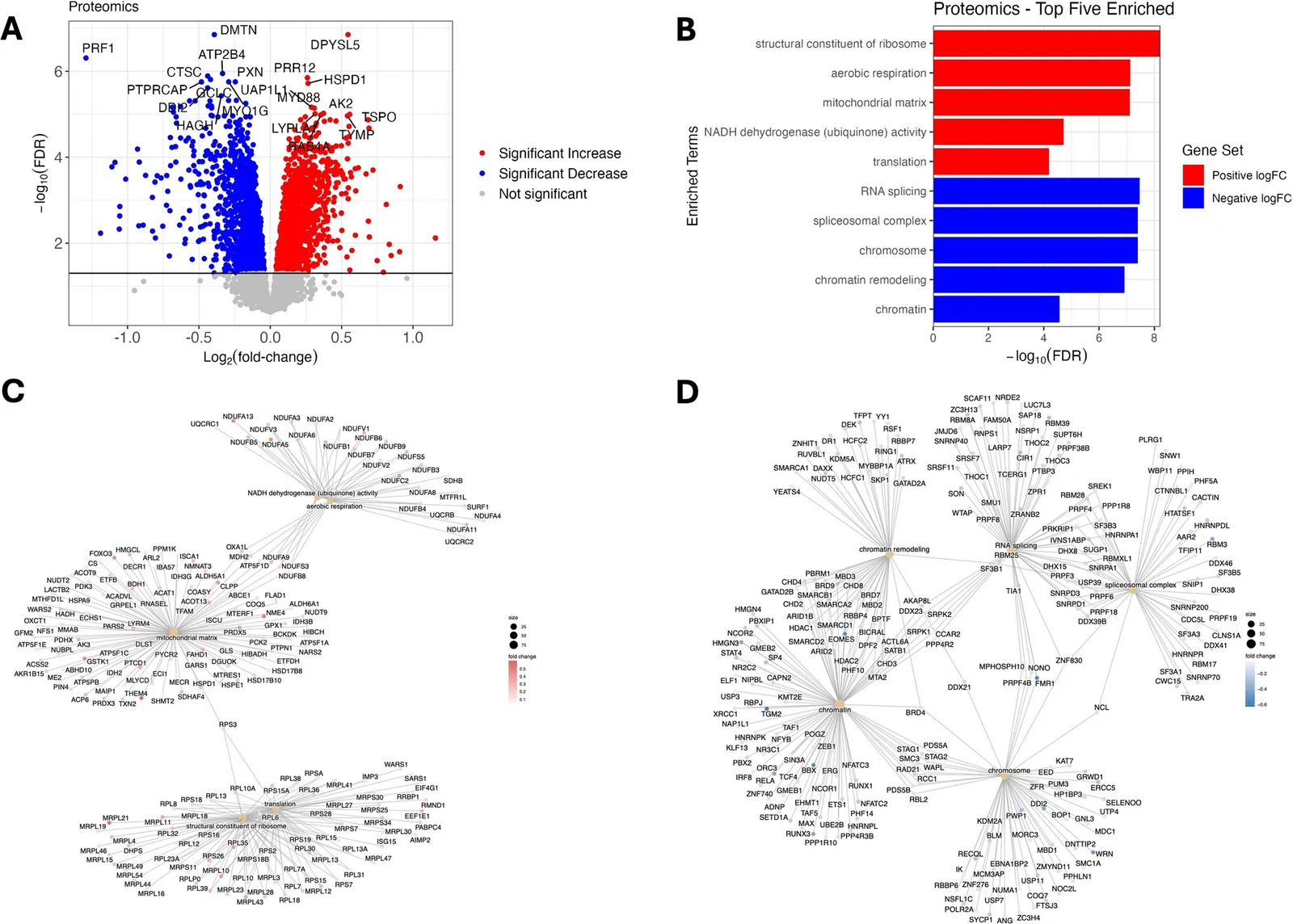
Proteomics data. **A** Volcano plot depicting differentially abundant proteins showing fold-changes (*x*-axis) and statistical significance (−log10(FDR), *y*-axis) with increased abundance in red and decreased abundance in blue (FDR < 0.05). **B** Bar chart showing top 5 enriched Gene Ontology (GO) pathways with increased (red) and decreased protein abundance (blue). **C** The gene-concept network (CNET) plot depicts the linkages between enriched pathway terms with proteins/protein identifiers annotated with the specific pathway term and represents them as a network. **D** CNET plot linking the proteins that were the most significantly decreased in abundance from GO pathways

A far stronger and disease-discriminating signal was observed in the proteomics layer, with a total of 2892 dysregulated proteins, 1370 proteins significantly increased (FDR *p*-value < 0.05), and 1522 proteins significantly decreased (FDR *p*-value < 0.05) in the RTT group compared to controls. As with the transcriptomics, proteins involved in the ribosomal machinery, protein synthesis, and mitochondrial function were upregulated. In addition, proteins involved in neural development, such as dihydropyrimidinase-like 5 (DPYSL5) and proline-rich protein 12 (PRR12), were highly upregulated. Interestingly, marked downregulation of proteins governing chromatin remodelling and chromosomal structure (ATRX Chromatin Remodeler; ATRX) and of proteins associated with immune signalling function (PRF1; Perforin) and cytoskeletal dynamics (DMTN; Dematin Actin Binding Protein and MYO1G; Myosin IG) was also evident (Fig. 4A). Pathway enrichment analysis found that, like the transcriptomics data, proteins with increased abundance in the RTT cohort were enriched in pathways related to ribosomal and mitochondrial functions. However, unlike the transcript data, proteins with decreased abundance were more enriched in pathways associated with RNA splicing and chromatin remodelling (Fig. 4B). CNET plots depict the GO pathways linked to proteins increased in individuals with RTT, including those involved in mitochondrial respiratory chain Complex I activity (NDUF), the mitochondrial matrix, and translation (Fig. 4C). A separate CNET plot depicts GO pathways linked to proteins decreased in individuals with RTT, including those involved in RNA splicing and chromatin function (Fig. 4D).

**Fig. 5:**
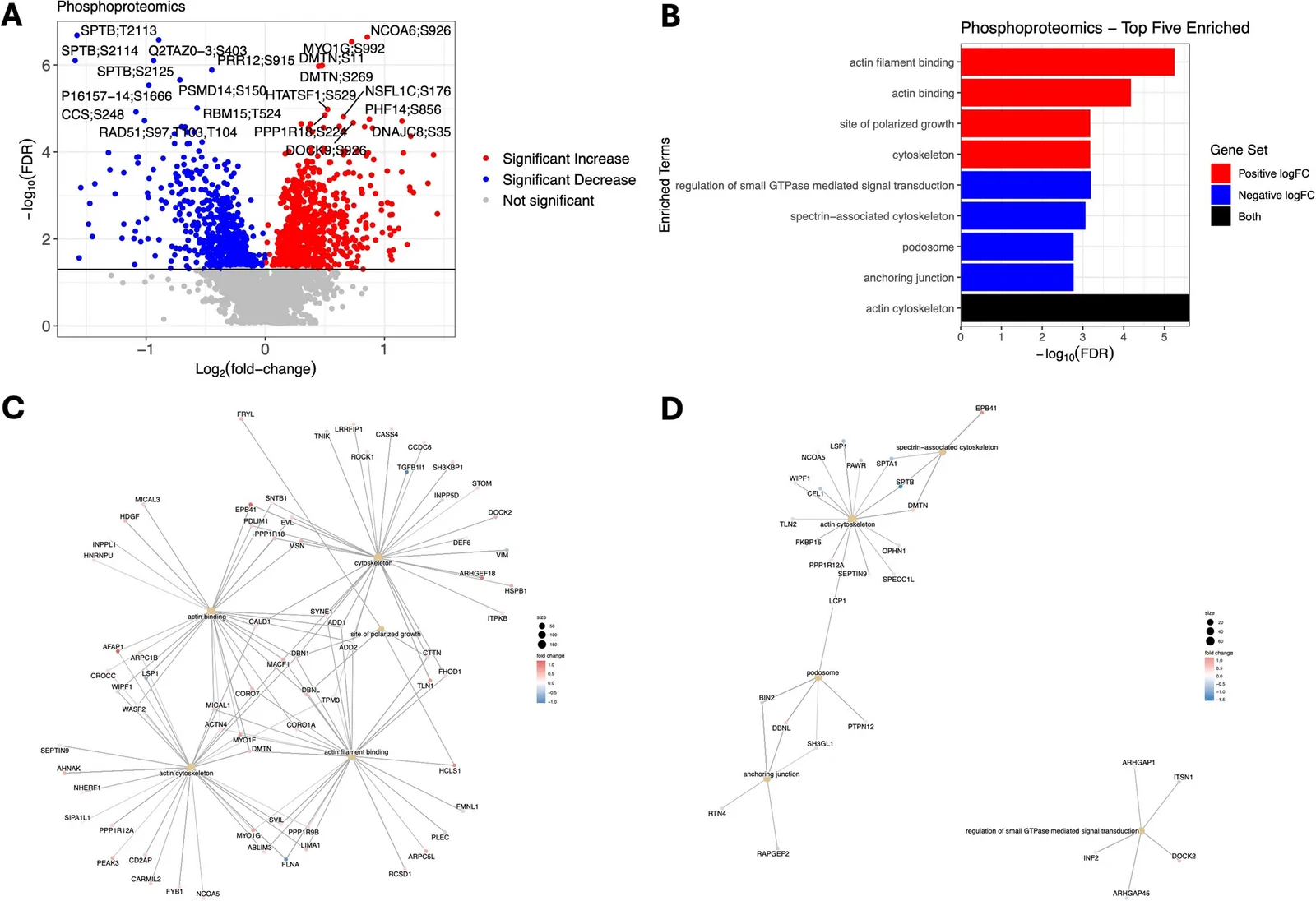
Phosphoproteomics data. **A** Volcano plot depicting differentially abundant phosphosites showing fold-changes (*x*-axis) and statistical significance (−log_10_(FDR), *y*-axis) with increased abundance in red and decreased abundance in blue (FDR < 0.05). **B** Bar chart showing top 5 enriched Gene Ontology (GO) pathways with increased (red), decreased phosphoprotein abundance (blue), and both increased and decreased phosphoprotein abundance (black). **C** The gene-concept network (CNET) plot depicts the linkages between enriched pathway terms and phosphoproteins, linking the significantly increased phosphoproteins from the GO pathways. **D** CNET plot linking the significantly decreased phosphoproteins from the GO pathways

Phosphoproteomic changes captured the dysregulated dynamic state of intracellular signalling and structural regulation in RTT cells. Of the 2324 dysregulated phosphosites, 1522 were downregulated, outnumbering the 802 upregulated phosphosites by nearly 2:1 (FDR < 0.05) (Fig. 5A). Enhanced cytoskeletal dynamics involving increased actin remodelling and filament organisation was evident by the upregulation of 2 phosphorylated sites (S11 and S269) in DMTN, a crucial cytoskeletal protein that binds, bundles, and stabilises actin filaments (also downregulated at the protein level) and at S992 in MYO1G, an actin-based motor protein that links the cytoskeleton to the plasma membrane. Increased phosphorylation sites were also observed on proteins associated with chromatin remodelling such as ATRX (S1082, Y1087, and S1348) and NCOA6 (S926), a transcriptional coactivator and chromatin regulator. The most striking example of low phosphorylation sites corresponded to spectrin beta (SPTB) at T2113, S2114, and S2125 sites, which reflects a broad suppression of cellular membrane scaffolding and structural stability as well as signal transduction. Of note, PRR12, which was increased at the protein level, showed significant reduction in phosphorylation at S915. Pathway enrichment (GO) analysis revealed that phosphoproteins with both increased (DMTN) and decreased abundance (SPTB) were enriched in pathways related to the actin cytoskeleton (Fig. 5B), a finding that was supported by the gene-concept network (CNET) analysis (Fig. 5C and D).

**Fig. 6:**
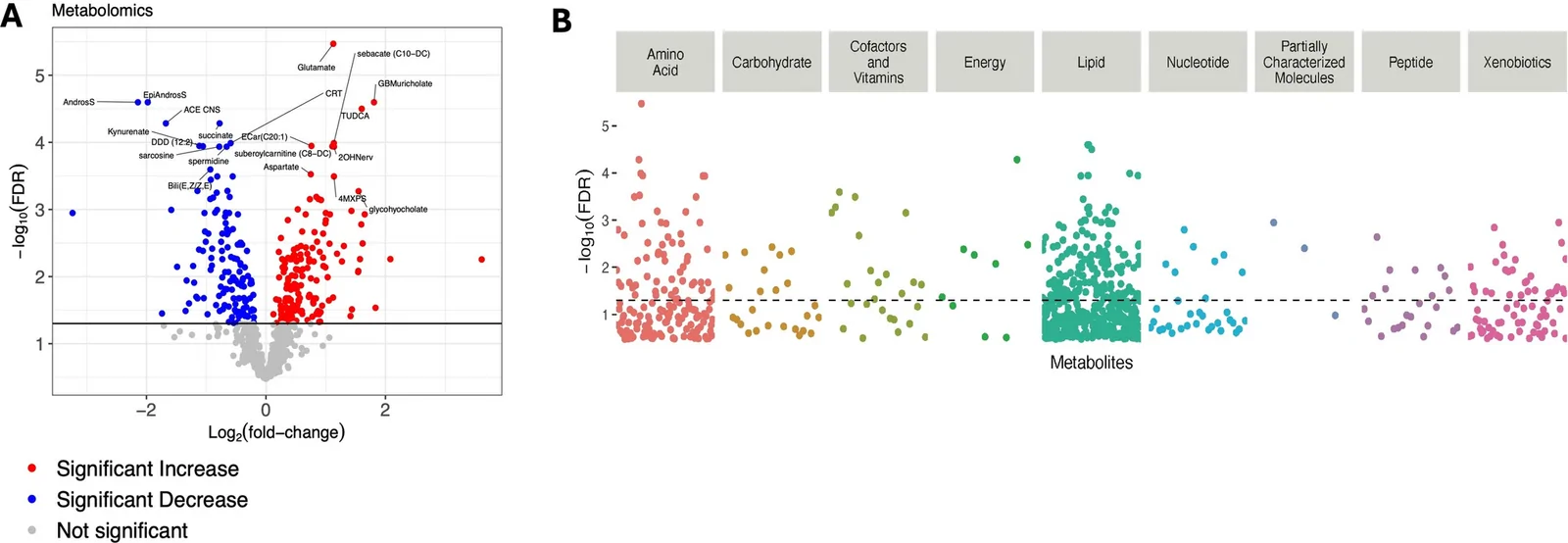
Metabolomics data. **A** Volcano plot depicting differentially abundant metabolites showing fold-changes (*x*-axis) and statistical significance (−log10(FDR), *y*-axis) where significantly increased metabolites are in red and significantly decreased metabolites are in blue (FDR < 0.05). 2OHNerv, 2-hydroxynervonic acid; 4MXPS, 4-methoxyphenol sulphate; ACE CNS, N‐acetylcarnosine; Bili(E,Z/Z,E), bilirubin (E,Z or Z,E); CRT, creatinine; AndrosS, androsterone sulphate; DDD(12:2), dodecanedioate; ECar(C20:1), eicosenoylcarnitine (C20:1); EpiAndrosS, epiandrosterone sulphate; GBMuricholate, glycol-beta-muricholate; TUDCA, tauroursodeoxycholate. **B** Manhattan plot displaying all metabolites characterised and ordered by chemical groups (*x*-axis) and statistical significance (−log10(FDR), *y*-axis) for each metabolite. The (black dashed) line of significance appears at a significance threshold of FDR = 0.05

Differentially expressed metabolites captured the metabolic consequences of MeCP2 deficiency in RTT cells and, like for transcriptomics, we observed a modest differential expression, with 108 being significantly increased and 102 significantly decreased (FDR < 0.05) (Fig. 6A). We identified increased excitatory energy-linked metabolites, such as glutamate and aspartate, as well as lipids and bile acids such as muricholate and TUDCA, alongside decreases in breakdown products (creatinine, kynurenate, glutamine degradant). Figure 6B shows a Manhattan plot grouping all observed metabolites by chemical category where a significant number of changes were observed in lipids, followed by amino acids, including glutamate. Pathway-level enrichment analysis using the Kyoto Encyclopedia of Genes and Genomes (KEGG) did not identify any pathway that remained significantly enriched after correction for multiple testing (all values had an FDR > 0.05).

### MOFA2 Latent Factor 1 Identifies a Disease-Associated Molecular Axis That Separates Rett Syndrome from Controls

**Fig. 7:**
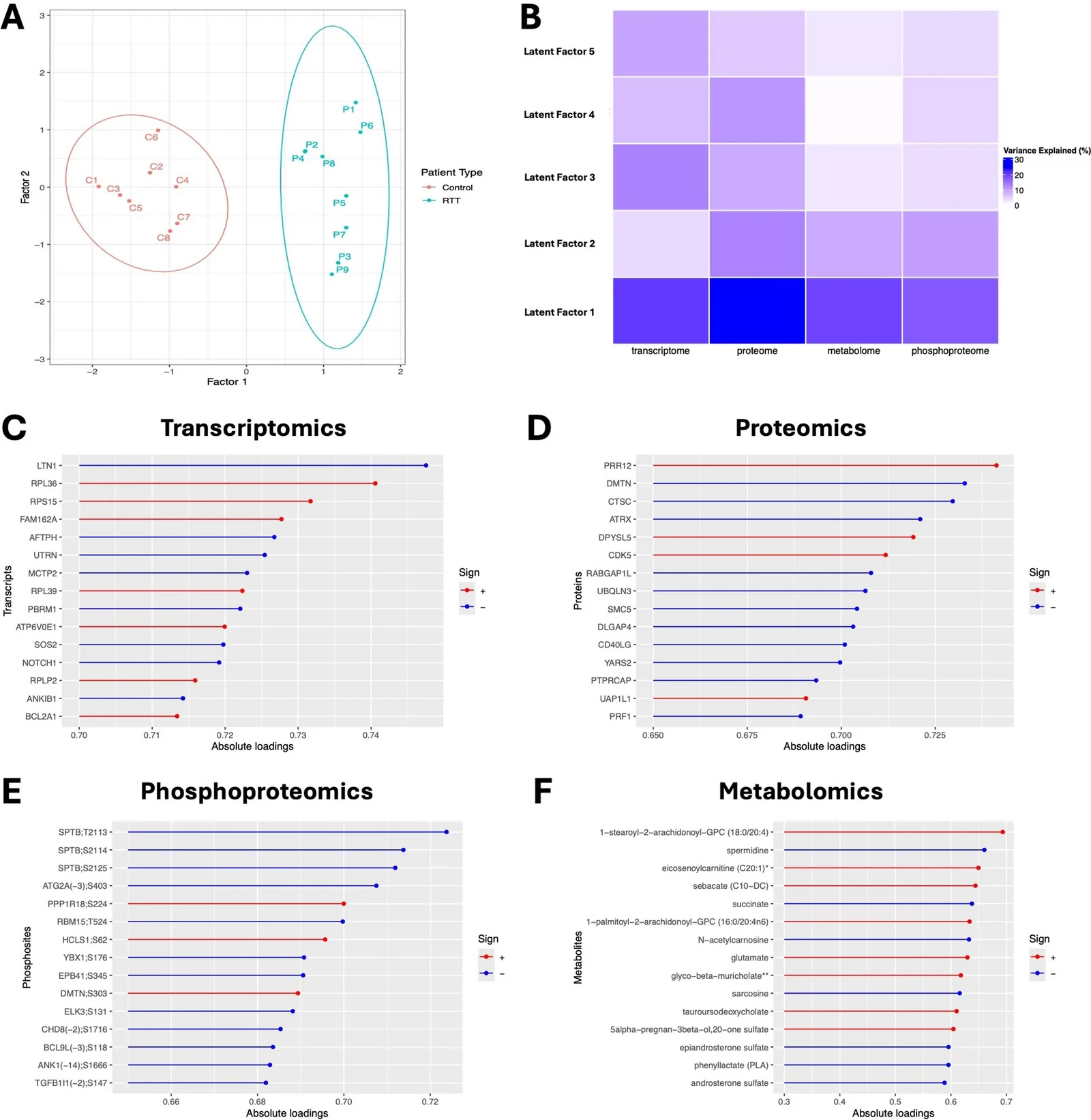
Omics (MOFA2) analysis. **A** MOFA2 sample-score plot showing separation of individuals with RTT and controls across Latent Factor 1 and Latent Factor 2. **B** Factor heat map displaying explained variance (%) delineated by each omics layer. **C** Line plot showing the magnitude of the absolute loadings (*x*-axis) of the top 15 transcripts (*y*-axis) from the transcriptomics dataset. **D** Line plot showing the magnitude of the absolute loadings (*x*-axis) of the top 15 proteins (*y*-axis) from the proteomics dataset. **E** Line plot showing the magnitude of the absolute loadings (*x*-axis) of the top 15 phosphosites (*y*-axis) from the phosphoproteomics dataset. **F** Line plot showing the magnitude of the absolute loadings (*x*-axis) of the top 15 metabolites (*y*-axis) from the metabolomics dataset. For **C**–**F**, positive (red) and negative (blue) contributions are indicated with colour and signs (+, −), with positive weights representing higher levels of expression in the RTT cohort and negative weights lower levels of expression in the RTT cohort

Rather than relying on a reductionist approach for biomarker discovery, we then investigated a holistic systems biology model to identify common factors underlying the cellular processes revealed by individual molecular layers. To do this, we used a robust, unsupervised machine-learning matrix factorization analysis tool, Multi-Omics Factor Analysis (MOFA2) [22, 40] to integrate multiple omics datasets from individuals with RTT and controls. MOFA2 factors are latent variables that capture the dominant sources of variation across the omics datasets. Each factor represents a distinct underlying signal, defined by its sample scores, feature loadings, and contribution from each omics modality. By modelling these latent factors, MOFA2 can identify coordinated or modality-specific molecular patterns and provides an integrated view of cross-omics relationships that are not detectable through single-omics analyses. MOFA2 Latent Factor 1 represents the primary ‘disease axis’, perfectly separating RTT from controls without overlapping confidence intervals as observed in the MOFA2 latent factor plot (Fig. 7A and Suppl. 1). The variance heatmap revealed that this axis is principally shaped by the proteome, followed by the transcriptome, as observed by the darker blue colours across all factors in this omics, indicating that the core RTT signature in blood is driven by transcript and protein expression (Fig. 7B). We then examined the absolute weightings (overall importance) of the top 15 features, by absolute Factor 1 loading, from each omics layer (Fig. 7C–F and Suppl. 2).

Based on the top weighted molecules in Latent Factor 1, we identified five significantly regulated biological cellular processes with shared biology across the omic datasets. These included (1) translational stress, (2) chromatin collapse, (3) cytoskeletal disorganisation, (4) immune dysfunction, and (5) metabolic gridlock.

- Translational stress: The MOFA2 transcriptomics positive loadings (Fig. 7C) were found to be enriched in upregulated ribosomal subunits (RPL36, RPS15, RPL39).
- Chromatin collapse: A suppression of chromatin remodelling and transcriptional regulation was suggested by the negative loading of ATRX, a major chromatin remodelling protein (Fig. 7D). This was supported by the upregulation of ATRX at three different sites in the individual phosphoproteomic data (Fig. 5A). Further support for chromatic collapse was provided by the negative loadings of the phosphosites of the chromatin/transcriptional regulators CHD8, BCL9L, and RBM15.
- Cytoskeletal disorganisation: The negative MOFA2 proteomic weightings (Fig. 7D) supported by the limma phosphoproteomic data (Fig. 5) suggest a disorganisation in prominent structural and membrane-associated targets (SPTB, DMTN, EPB41, DLGAP4). Notably, spectrin beta (SPTB) showed decreased phosphorylation at three sites (Fig. 7E), and DMTN protein expression was negatively loaded (Fig. 7D) and positively loaded in the phosphorylation of residue S303 (Fig. 7E).
- Immune dysfunction: Reduced immune cell activation was supported by the negative proteomic weightings of proteins such as Perforin (PRF1) and CD40LG (Fig. 7D). Both proteins are involved in the adaptive immune response where PRF1 is produced by CD8 cells and CD40LG is expressed on activated CD4 cells.
- Metabolic gridlock: A systemic metabolic dysfunction in glutamatergic and bile acids metabolism suggested by positive loadings of glutamate and muricholate (Fig. 7F) was supported by individual metabolomics showing increased levels of glutamate, aspartate, muricholate, and tauroursodeoxycholate (TUDCA) and reductions in succinate, creatinine, and glutamine degradant (Fig. 6A).

### Differential Correlation Network Analysis of MOFA2 Provides a Basis for a Novel Biomarker Signature

**Fig. 8:**
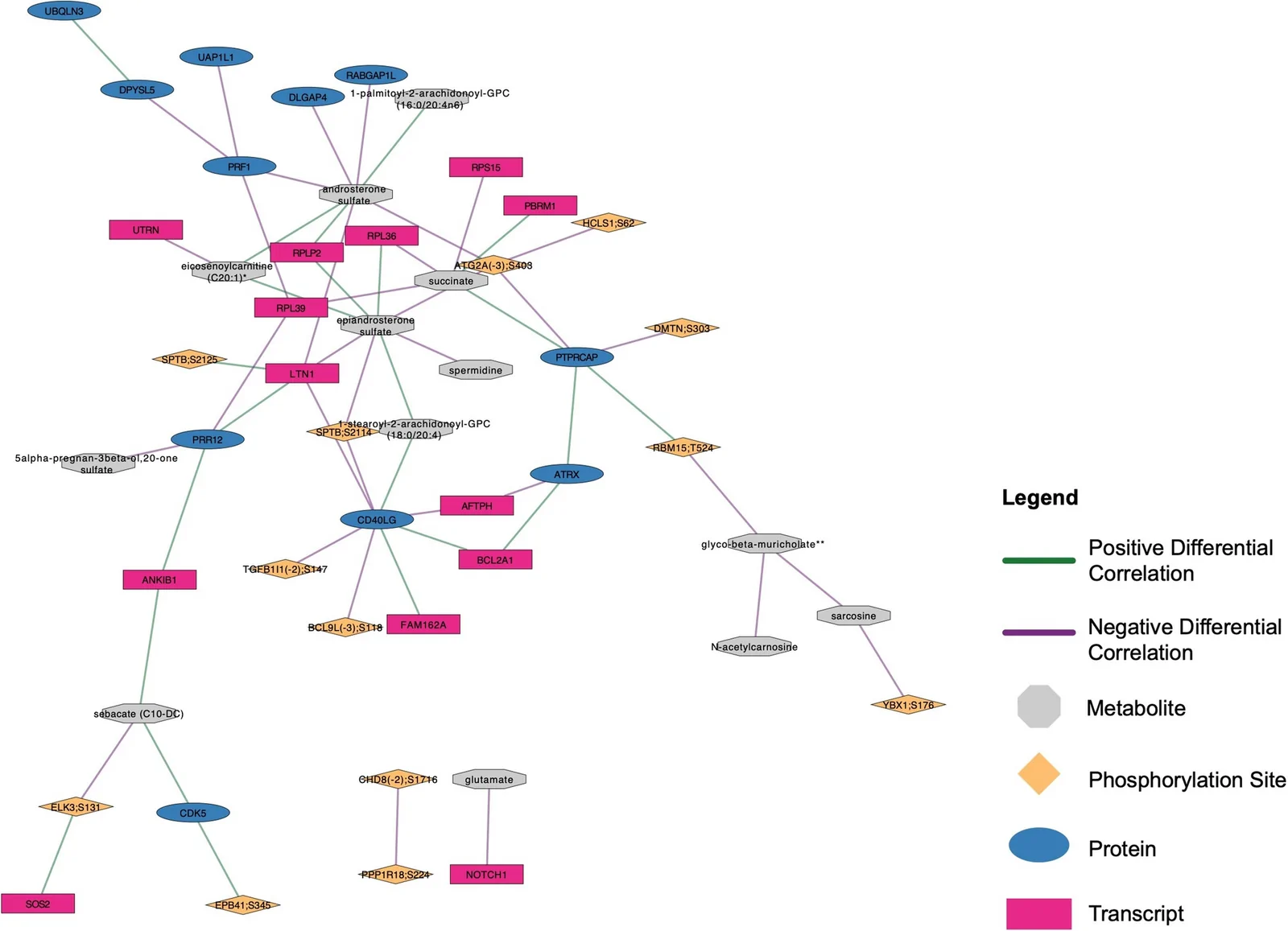
Differential correlation network analysis of significant MOFA2 molecules. Differential correlation network analysis of significant dysregulated molecules shows significant changes in their functional relationships in individuals with RTT versus controls. The nodes represent molecules from different omics analyses (pink squares, transcripts; blue ovals, proteins; yellow diamonds, phosphosites; grey octagons, metabolites). The edges (lines) indicate significant differential correlations (|Δcorrelation| > 1, *p* < 0.05 in either individuals with RTT or control samples). The green edges (lines) represent stronger positive correlations in RTT, and purple edges (lines) represent stronger negative correlations in RTT compared to controls

To visualise the interaction between the various omics datasets, a network-based approach was utilised (Fig. 8). The network is comprised of nodes that were generated from analysing the Pearson correlations between the top 15 features, by absolute MOFA2 Factor 1 loadings, from each omics layer. The network connects nodes across all four omics layers, acting as a structural map of the disease state, confirming that RTT is characterised not just by changes in molecular abundance but also by profound molecular rewiring (Fig. 8).

The green lines between molecules indicate a stronger positive correlation in RTT; i.e. those molecules are more positively correlated in individuals with RTT than in controls, suggesting that the abundance of these molecules is in tandem compared to their relationship in the control population. Conversely, the purple lines indicate stronger negative correlations in RTT; i.e. the correlation between the two molecules is weaker in individuals with RTT than in controls.

The network is organised into distinct clusters, indicating groups of molecules that are co-regulated. A number of key nodes include those involved in mitochondrial metabolism such as eicosenoylcarnitine (C20:1-carnitine) and succinate, immune signalling and secretory functions (PRF1, CD40LG, PTPRCAP), ribosomal dysregulation (LTN1), chromatin remodelling (ATRX), and cytoskeletal organisation (SPTB phosphorylation).

### MOFA2 Latent Factor 2 Identifies a Severity-Associated Molecular Axis That Correlates with Clinical Severity

**Fig. 9:**
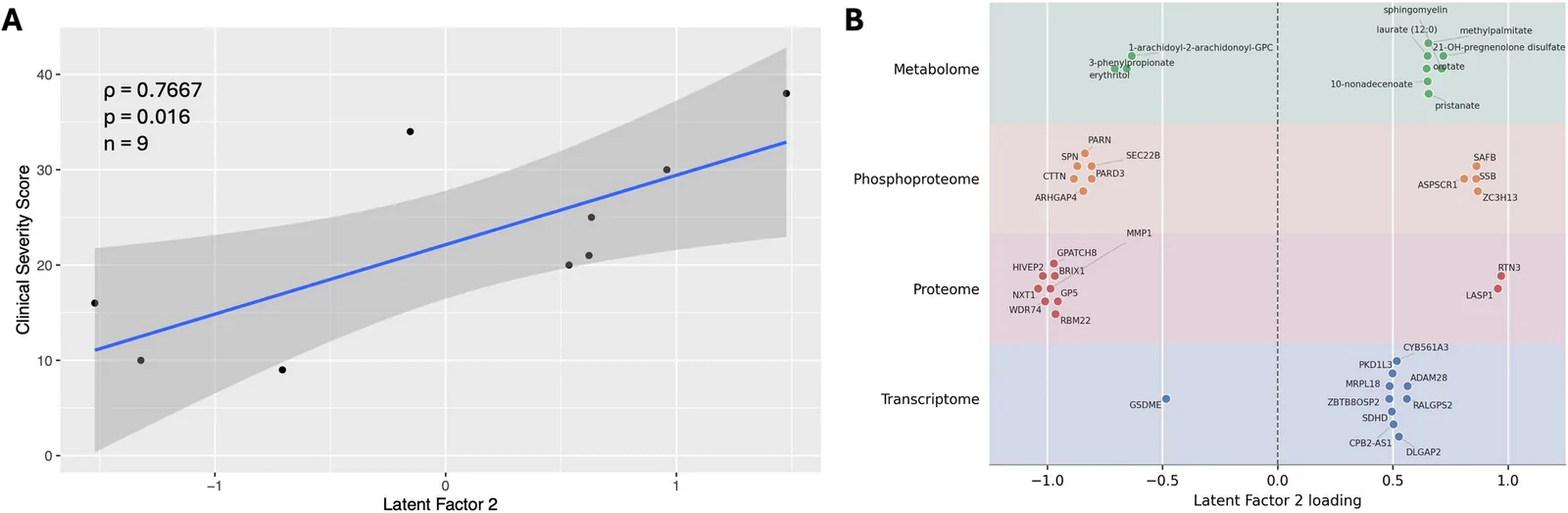
Factor 2 (severity-associated) analysis. **A** MOFA2 Factor 2 shows a positive correlation with clinical severity (CSS) of the RTT individuals (Spearman’s *ρ* = 0.7667, *p* = 0.016, *n* = 9). **B** Scatter plot showing the top 10 features with the highest loading scores on Latent Factor 2 from each omics layer (metabolome, phosphoproteome, proteome, and transcriptome), colour-coded by layer. The *x*-axis shows the assigned Factor 2 loading for each feature. Consistent with the positive correlation between Factor 2 and clinical severity shown in **A**, features with positive loadings (right of the dashed line) are associated with higher clinical severity, while features with negative loadings (left of the dashed line) are associated with lower clinical severity

To explore additional implications of our data, we ran a correlation analysis between Latent Factor 2 and CSS scores of individuals with RTT. Indeed, Latent Factor 2 showed a significant positive correlation with the CSS (Spearman’s *ρ* = 0.77, *p* = 0.016) (Fig. 9A). This linear relationship indicates that higher disease severity scores are associated with higher weights for Latent Factor 2 suggesting that the molecular features with high absolute weighting scores on Factor 2 capture within-disease variation and, therefore, represent a candidate molecular signature of disease severity. We then investigated the significantly dysregulated molecules identified in Latent Factor 2 and observed that the top ranked molecules were involved in cellular communication, energy metabolism, and gene regulation (transcriptomics); regulation of cytoskeletal dynamics and signalling, neuronal growth and connectivity, and protein homeostasis, including endoplasmic reticulum stress and unfolded protein response pathways (proteomics); actin cytoskeleton (phospho-proteomics); and fatty acid and metabolic homeostasis (metabolomics) (Fig. 9B, Suppl. 1).

Of the Latent Factor 2 molecules, highly positive weighted molecules, such as sphingomyelin, which is ranked 2nd among metabolites (+ 0.71), highly abundant in the central nervous system, particularly in myelin sheaths and RTN3 (reticulon 3), a protein which shapes endoplasmic reticulum tubules and regulates membrane dynamics and is highly expressed in the brain, which is ranked 6th among proteins (+ 0.96), are likely indicators of higher disease severity (Fig. 9B). In contrast, highly negatively weighted molecules, such as MMP1 (matrix metallopeptidase 1), a secreted zinc-dependent endopeptidase that breaks down interstitial collagen in the extracellular matrix, which is ranked 4th among proteins (− 0.98), could serve as markers of lower disease severity (Fig. 9B).

### Orthogonal Validation Confirms Increased Circulating Glutamate

**Fig. 10:**
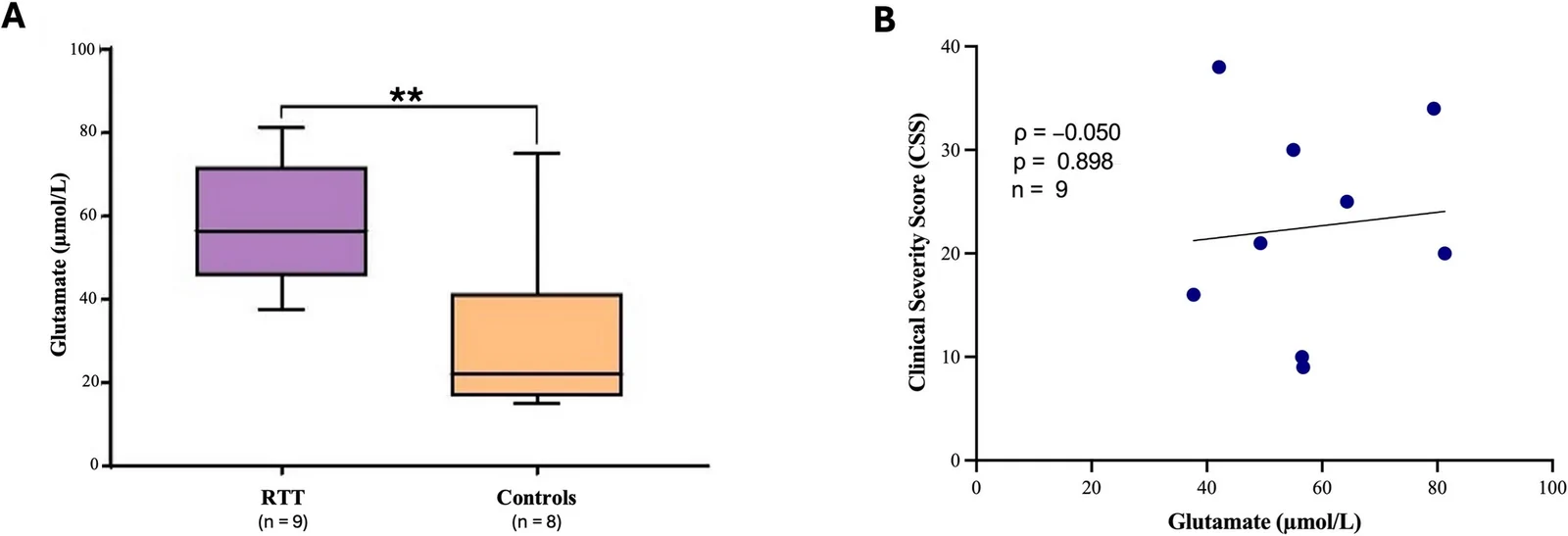
Individuals with RTT have increased circulating glutamate levels relative to controls. **A** UPLC-UV plasma glutamate concentrations are graphed as box and whisker plots. Individuals with RTT are depicted as purple and age- and sex-matched controls depicted as orange. Statistical comparisons between the affected individuals and unaffected controls were made by the Mann Whitney *U* test. **p < 0.01. **B** Spearman’s coefficient correlation graph showing no correlation between glutamate levels and CSS Spearman’s *ρ* = − 0.050, *p* = 0.898, *n* = 9)

As glutamate was the highest dysregulated metabolite in RTT, we used UPLC-UV to orthogonally validate the glutamate dysregulation observed in the individual metabolomics limma and MOFA2 analyses. We found a significant increase in circulating blood glutamate concentrations in the RTT cohort compared to the age- and sex-matched controls in this study (Fig. 10A and Table 2). We then tested whether individuals’ glutamate levels were related to disease severity but found no correlations with CSS (Fig. 10B), suggesting that glutamate is a biomarker of disease but not of severity.

**Table 2:** Blood glutamate concentrations analysed by UPLC-UV and corresponding CSS in individuals with Rett syndrome and age- and sex-matched controls

ID	Patient type	Glutamate (µmol/L)	CSS
C1	Unaffected	21.4	N/A
C2	Unaffected	28.6	N/A
C3	Unaffected	75.0	N/A
C4	Unaffected	14.9	N/A
C5	Unaffected	45.7	N/A
C6	Unaffected	21.9	N/A
C7	Unaffected	15.3	N/A
C8	Unaffected	22.3	N/A
P1	RTT	42.1	38
P2	RTT	64.3	25
P3	RTT	56.5	10
P4	RTT	49.3	21
P5	RTT	79.4	34
P6	RTT	55.0	30
P7	RTT	56.7	9
P8	RTT	81.3	20
P9	RTT	37.7	16

## Discussion

Rett syndrome is a complex neurodevelopmental disorder with neurological and metabolic manifestations. Although the disorder is primarily caused by pathogenic variants in *MECP2*, the downstream effects of MeCP2 dysfunction are unlikely to be captured by a single molecular measurement. This has made the identification of molecular biomarkers of RTT challenging, particularly biomarkers that reflect disease state, clinical severity or biological response to emerging therapies. While MeCP2 is most extensively studied in the central nervous system, it is also expressed beyond the brain, and RTT is increasingly recognised as a multisystem disorder. Peripheral blood, therefore, represents a biologically plausible compartment in which to detect disease-associated molecular changes, more than simply a convenient sampling source. In this study, we used an integrated blood-based multi-omics approach, incorporating PBMC transcriptomics, proteomics, and phosphoproteomics and plasma metabolomics, to identify molecular signatures associated with RTT. Rather than identifying one dominant molecule or pathway, using a machine learning bioinformatic approach, our findings indicate that RTT is associated with a complex pattern of abnormalities involving multiple cellular processes across several molecular layers. These included pathways related to ribosomal and translational function, mitochondrial and energetic processes, chromatin and RNA-processing regulation, cytoskeletal phospho-regulation, immune and secretory features, and plasma lipid/cholesterol metabolism and energy-linked amino acids. These findings suggest the presence of peripheral molecular signatures associated with disease-state and phenotypical variability in RTT. Although alterations in some of these processes have been previously reported in RTT, our converging multi-omics data strengthens the biological relevance and potential specificity of the identified molecular signatures of RTT disease state and progression.

A key finding of this study is that RTT-associated molecular differences were most informative when interpreted as interconnected pathway-level changes rather than as independent omics modalities. Comparisons between RTT and control groups identified changes within each molecular layer, while MOFA2 provided an integrated framework for determining shared sources of variation across the datasets. MOFA2 Latent Factor 1 separated individuals with RTT from controls, supporting the presence of an integrated RTT-associated molecular network in peripheral blood. This latent factor was not restricted to a single modality; although transcriptomic and proteomic features contributed strongly, phosphoproteomic and metabolomic features also added to the integrated signal. This suggests that the RTT blood signature spans gene expression, protein abundance, post-translational regulation, and systemic metabolic layers, rather than being driven by a single omics modality. In addition, MOFA2 Latent Factor 2 identified a severity-associated molecular network, which correlated with CSS scores, with a potential for predicting disease severity in individuals with RTT.

The cellular processes identified by individual omics analysis as differential in individuals with RTT include ribosomal and translation functions, mitochondrial metabolism, chromatin remodelling, immune signalling and secretory function, cytoskeletal organisation and lipid and cholesterol handling pathways, and energy-linked amino acids. These are discussed in the following sections, in the context of their coordinated variation and interactions across molecular layers.

### Ribosomal and Translational Stress Represents the Strongest Cross-Layer Signal

The most consistent cross-molecular layer finding involved ribosomal and translation-associated function. Both transcriptomic and proteomic enrichment analyses identified ribosomal pathways to be elevated in individuals with RTT, and MOFA2 loadings further highlighted ribosomal transcripts including RPL36, RPL39, RPLP2 and RPS15, and RPS16 and RPS19 as contributors to the integrated disease-associated factor (Latent Factor 1). This convergence across transcriptomic, proteomic, and integrative analyses suggests that altered ribosomal or translational machinery is an internally consistent feature of the RTT-associated peripheral signature. These findings are consistent with previous reports of altered translational regulation in RTT models, including reduced AKT/mTOR signalling and associated protein synthesis dysregulation in Mecp2-mutant mice, and altered ribosome engagement in RTT patient-derived neurons [41, 42]. Increased abundance of ribosomal transcripts and proteins may reflect altered cellular homeostasis, adaptive remodelling of the protein synthesis machinery, or a compensatory response to chronic cellular stress. Upregulated genes were heavily concentrated in the ribosomal structure category indicating a transcriptional push towards a globally increased protein synthesis machinery. Importantly LTN1 which encodes listerin E3 ubiquitin protein ligase 1, an E3 ubiquitin ligase that acts in ribosome quality control to clear stalled translation products [43], was a major hub in the differential correlation network (Fig. 8). Translational dysregulation has previously been reported in mouse and patient-iPSC derived models, thus supporting this core observation of a defective protein synthesis machinery in RTT [41, 42].

### Mitochondrial and Bioenergetic Changes may Reflect Cellular Adaptation Rather Than Enhanced Mitochondrial Function

Mitochondrial and bioenergetic pathways also emerged as a major theme, particularly in the transcriptomic and proteomic datasets. Genes and proteins associated with mitochondrial energy production, respiratory chain function, mitochondrial matrix biology, and NADH dehydrogenase activity were enriched among features increased in RTT. These findings are aligned with the multiple studies describing mitochondrial dysfunction in RTT and may reflect a RTT-associated bioenergetic adaptation and compensatory response to impaired mitochondrial efficiency, altered energetic demand, oxidative stress, or broader cellular stress [11, 12, 16]. Importantly, succinate, an intermediate of the tricarboxylic acid (TCA) cycle, which plays a crucial role in adenosine triphosphate (ATP) generation in mitochondria, was negatively loaded onto the metabolomics component of MOFA2 Latent Factor 1 and was a hub molecule in the differential correlation network (Fig. 8). Together, these findings position succinate as a key metabolic node linking mitochondrial dysfunction with immune activation and inflammatory signalling, thereby providing a plausible mechanism through which altered bioenergetics may contribute to cellular activation and systemic inflammation in RTT [44].

### Chromatin and RNA-Processing Features Provide a Biologically Plausible Link to MeCP2-Associated Regulation

Chromatin regulation and RNA-processing pathways provide one of the most biologically plausible links between the peripheral multi-omics signature identified in this study and known molecular functions of MeCP2. The proteomic analyses found decreased abundance of proteins involved in chromatin remodelling, chromosome organisation, and RNA splicing such as the major chromatin remodeller, ATRX, which was supported by the increased phosphorylation sites in ATRX at S1082, Y1087, and S1348.

MOFA2 loadings validated these findings with ATRX negatively loaded in the proteomics and the chromatin/transcriptional regulators CHD8, BCL9L, and RBM15 to be negatively loaded in the phosphoproteomics. The MOFA2 analyses highlighted the dynamic state of these processes, involving phosphorylation, and given MeCP2’s established role as a chromatin-associated methyl-DNA reader and transcriptional regulator [45, 46], these findings are relevant to RTT biology and suggest a suppression of the epigenetic machinery in peripheral blood cells [16, 47]. Given the critical role of MeCP2 in epigenetic regulation [48], this may reflect the primary consequence of MeCP2 dysfunction and a broader failure or suppression of the epigenetic and chromatin-remodelling apparatus in RTT cells.

### Proteomic and Phosphoproteomic Analyses Reveal Cytoskeletal Disorganisation

Dysregulation of pathways involved in cytoskeletal function has previously been identified in RTT [15, 49, 50]. Additionally, alterations in the neuronal cytoskeleton thought to be contributing to distinctive cortical dendritic anomalies have been previously reported in postmortem samples from individuals with RTT [49]. The individual omics data showing a downregulation in proteins such as DMTN and MYO1G was supported by the downregulation of phosphorylation sites of SPTB and upregulation of phosphorylation sites on DMTN*.*

While there was evidence of cytoskeletal deficits across several molecular layers in this study, the most striking evidence came from the prominent negative proteomic and phosphoproteomic MOFA2 loadings for structural and membrane-associated targets, especially SPTB and DMTN*.* Interestingly, 3 different phosphorylation sites on SPTB were negatively loaded and the phosphorylation of DMTN was positively loaded while its overall protein expression was negatively loaded. SPTB is a major component of red blood cell membranes where it forms a flexible scaffold that acts as a membrane skeleton [51]; thus, the profound decrease in phosphorylation sites suggests an alteration of the cellular cytoskeletal architecture in RTT. DMTN is an actin-binding and -bundling protein that, while it has a relatively high brain expression, is known to also play a structural role in erythrocytes. DMTN’s high levels in blood are linked to the protein phosphorylation-regulated actin-bundling activity [52]. However, the role of DMTN and SPTB in the brain pathology of RTT is uncertain. Thus, these findings suggest altered regulation of membrane-associated cytoskeletal proteins in RTT blood cells supporting a cytoskeletal phospho-regulatory signature that may reflect altered cell structure, signalling, activation state, or immune-cell behaviour.

In addition, we observed RTN3 to be ranked highly positive in the Latent Factor 2 analysis and correlating with high disease severity (Fig. 9B). RTN3 is an endoplasmic reticulum membrane-shaping protein involved in membrane dynamics and intracellular trafficking. Given the close functional coupling between the endoplasmic reticulum and the cytoskeleton, the concurrent alterations in RTN3, SPTB, and DMTN suggest a disruption of an endoplasmic reticulum-membrane cytoskeletal network in RTT.

### Immune and Secretory Features Suggest Altered PBMC-Associated Immune Biology in RTT

A downregulation of immune signalling was apparent in the individual omics evidenced by a downregulation in proteins such as PRF1 and PTPRCAP. However, a pronounced downregulation in immune signalling and secretion was revealed predominantly by MOFA2 proteomic profiles characterised by PRF1 and CD40LG negative loading, in addition to *PTPRCAP* as a hub gene in the differential correlation network. PRF1 is central to cytotoxic lymphocyte function through the perforin-granzyme pathway [53], while CD40LG contributes to T-cell-dependent immune signalling and communication between immune-cell populations [54] and PTPRCAP is linked to lymphocyte activation [55]. Immune and inflammatory pathways have been implicated in RTT models, including evidence of sustained NF-κB signalling in MeCP2-deficient myeloid-derived cells and aberrant NF-κB signalling in Mecp2-null mice [56, 57]. Reduced abundance of immune-effector molecules could reflect altered expression within immune cells as well as differences in PBMC cell-type composition, such as altered proportions of cytotoxic T cells, NK cells, or monocytes.

The negative ranking of MMP1 in the Latent Factor 2 analysis supports these observations. Being a matrix metalloproteinase, MMP1 is involved in both extracellular matrix remodelling and immune regulation and, beyond its role in collagen degradation, MMP1 influences cytokine signalling, leukocyte migration, and inflammatory responses. The association of MMP1 with lower disease severity may, therefore, reflect preservation of immune and tissue homeostatic processes that become disrupted with increasing RTT severity.

### Plasma Metabolomics Identifies Systemic Lipid/Cholesterol and Energy-Linked Amino Acid Changes

Metabolomics data provide a systemic biochemical perspective that complements the PBMC-based transcriptomic, proteomic, and phosphoproteomic findings. Plasma metabolomics identified alterations in amino acid-, lipid-, bile acid-, and cholesterol-related metabolites. To date, relatively few studies have examined circulating metabolomic or lipidomic signatures in RTT. Prior work has reported plasma metabolic differences between individuals with RTT and unaffected siblings, alterations in sphingolipid metabolism in blood/plasma samples, and changes in the CSF and plasma lipidome of individuals with RTT [14, 58, 59]. Altered cholesterol and lipid-related metabolites are consistent with previous reports implicating changes in lipid and bile acid expression and cholesterol metabolism in RTT, including studies of peripheral cholesterol profiles, patient-derived fibroblasts, and CSF/plasma lipidomics [59, 60].

In the individual metabolite analysis, glutamate, aspartate, TUDCA, and muricholate were highly upregulated and succinate downregulated. These findings were supported by a positive loading of glutamate and muricholate and a negative loading of succinate in the MOFA2 analysis, with orthogonal validation using UPLC-UV supporting the untargeted metabolomics finding of elevated glutamate. Higher glutamate levels have previously been reported in RTT in blood [14] and cerebrospinal fluid studies [61, 62], and MeCP2-deficient myeloid-derived cells have been shown to exhibit increased glutamate release at baseline and after stimulation [56]. In addition, in vitro studies using a MeCP2-deficient PBMC model [56, 63] showed increased glutaminase expression. Both elevated glutamate release and glutaminase expression would contribute to higher levels of glutamate in the bloodstream, as well as the central nervous system and peripheral tissues. Interestingly, increased glutaminase mRNA levels in mouse microglial cultures were suppressed by NF-κB inhibition [56] of relevance to the aforementioned NF-κB overactivity in RTT [56, 57]. Hence, the mechanism underlying higher glutamate levels may be related to excessive inflammation and immune dysregulation. While the validated individual glutamate concentrations in the participants with RTT were higher than those of the controls in our cohort, they were within the normative reference range (glutamate 14–113 µmol/L; NSW Biochemical Genetics Service, Westmead, Australia). Although this reference population is not age- or gender-matched to our cohort, the overlap in range of values should be taken into account when contemplating glutamate as a molecular disease-state biomarker. However, the use of glutamate levels as a longitudinal monitoring biomarker within individuals with RTT may still be considered. Studies that obtain baseline levels of amino acids prior to the commencement of a therapeutic intervention may help further elucidate the role of amino acid alterations in RTT.

As the metabolomic analysis was performed in plasma, this provides evidence of a circulating systemic signature rather than direct measures of PBMC-intrinsic biology and reflects contributions from multiple factors, including liver and gut function, diet, medication exposure, endocrine state, mobility, inflammation, or other systemic processes. Metabolomic profiles and MOFA2 loadings also highlight an increase in bile acids/cholesterol derivatives (glycocholate) and complex lipids, paired with other metabolites such as aspartate, dodecanedioate, eicosenoylcarnitine, and creatinine, which suggest a compensatory response to the aforementioned translation dysfunction and reflect a downstream effect to MeCP2 deficit. Reassuringly, many dysregulated metabolites identified in our study were also reported by Neul and colleagues including elevations in glutamate and decreases in arginine, N-acetyl carnosine and 3,4-hydroxyphenyl lactate [14], thus validating our findings. It is promising that these dysregulated molecules are consistent across both cohorts of patients, as this provides further support for pathogenic metabolic processes underlying the pathophysiology of RTT. However, it is important to note that both studies utilised the same wet-lab and initial bioinformatic platform for sample processing. Reproduction of the same findings on different analytical platforms is needed to provide ancillary confirmation.

In support of the potential of metabolomics data, we observed sphingomyelin to be ranked highly positive in the Latent Factor 2 analysis putting this lipid as a plausible candidate of disease severity (Fig. 9B). Sphingolipid metabolism has been previously reported to be perturbed in RTT CSF and plasma [58, 59]. While the direction of sphingomyelin change differs between cohorts and biological compartments (CSF versus plasma), these studies collectively support dysregulation of membrane lipid and cholesterol-associated pathways as a feature of RTT.

### Informing Future RTT Biomarkers: Disease State and Clinical Severity

Based on the integrated individual omics data into MOFA2 Latent Factor 1 and 2, we can identify a number of biologically robust, clinically relevant molecules, reflective of underlying disease mechanisms that may inform future RTT biomarkers.

- Translational stress: LTN1: Many ribosomal genes and proteins were found to be dysregulated in the individual omics layers, as well as in the MOFA2 transcript positive loadings. One gene of interest is *LTN1*, which we identified as central hub in the differential correlation network. However, rather than individual genes/proteins, we suggest a number of these molecules could be used in the form of a biomarker signature.
- Chromatin collapse: ATRX/CHD8: These molecules represent chromatin/epigenetic repression dysregulation in RTT supported by the coordinated downregulation of ATRX protein levels and decreased CHD8 phosphorylation. As MeCP2 is known to be an epigenetic reader, this suggests a systemic collapse of chromatin remodelling and a direct mechanistic consequence of the pathophysiology of RTT.
- Cytoskeletal dysregulation: SPTB/DMTN: SPTB, a spectrin-actin cytoskeleton biomarker has high biomarker potential since, as a constituent of the critical meshwork under the plasma membrane of red blood cells, its profound decrease in phosphorylation may signify a reduction in membrane structural integrity in RTT. With SPTB dominating the top negative MOFA2 phosphoproteomic loadings and DMTN representing the most negatively regulated protein, there is support for a strong systemic disease correlate reflecting cellular cytoskeletal stress of blood cells.
- Immune dysfunction: PRF1/CD40LG/PTPRCAP: These immune signalling and secretory molecules are critical for immune cell function and immune-synapse formation, and their suppression hints at functional immunodeficiency in RTT blood cells. Their top negative MOFA2 proteomics loadings are supported by decreased PRF1 protein expression in single omics. They have a potential as systemic dynamic biomarkers.
- Metabolic gridlock: Glutamate: Systemic increases in glutamate, which were orthogonally validated, and decreased glutamine degradants place glutamate as an attractive biomarker as it is highly measurable in standard plasma draws and reflective of systemic metabolic stress and immune dysfunction.

Based on our differential correlation network comparing omics data from RTT and controls, a number of nodes were identified with significant differential correlation, some showing positive correlation and others negative correlation, highlighting the interactions between many molecules and the important changes in functional relationships in individuals with RTT when compared with controls affecting among others the aforementioned candidate biomarkers. These molecular network findings not only elucidate a unique omics molecular signature but also provide important insights into RTT pathophysiology.

We conclude that a decrease in MeCP2 function induces a systemic molecular stress response that is detectable in peripheral blood and manifests as coordinated dysregulation of translational control, chromatin organisation, cytoskeletal architecture, immune function, and energy metabolism. Our integrated multi-omics analyses revealed that PBMC’s LTN1, ATRX, CHD8, SPTB, DMTN, PRF1, CD40LG, and PTPRCAP and plasma glutamate are the most promising disease-state (complementary diagnostic) candidate biomarkers while sphingomyelin, RTN3, and MMP1 appear to be the most suitable biomarkers of disease severity. Other components of the most informative latent factors could also be considered individually or in groups in future studies.

## Limitations and Future Directions

As with all rare conditions, the generalisability of our study is constrained by the small cohort size. While our data show a clear molecular signature, future replication studies involving larger cohorts of individuals with RTT, especially studies stratified by genotype or clinical severity, would be essential for the further development of the candidate biomarkers. Future collection of longitudinal data will also be critical in determining the stability of the reported latent factors and their potential to inform about the evolution of RTT and response to interventions. Future analyses of relationships between candidate biomarkers and clinical severity will need to incorporate more dynamic measures than the CSS [7]. Moreover, addition of samples from individuals with other neurodevelopmental disorders will be important for establishing the specificity of the reported molecular signatures. In addition, in a clinical setting, it is often difficult for patients to fast for prolonged periods of time, and this is particularly likely to have had an impact on the metabolomics component. Therefore, future work would benefit from analysing fasted samples (or correcting for this during analysis by including a comprehensive diet and medication summary). Additionally, it is likely that non-truncating variants in *MECP2* will have variable impacts on MeCP2 function, given that different domains have varying effects on transcription, often impacting different downstream pathways. Omics studies in tissues from individuals with RTT and the same genotype would provide better insights into downstream functional impacts relevant to specific patient subgroups.

Despite the aforementioned shortcomings, the consistency of the molecular processes identified across independent omics platforms supports the robustness of the integrated molecular signatures with potential to serve as complementary diagnostic measures or pharmacodynamic biomarkers. Integration of peripheral blood datasets with patient-derived neuronal models, including induced pluripotent stem cell-derived neurons and cortical organoids, will further strengthen the biological interpretation of these molecular signatures and help in characterising RTT pathophysiology by increasing our understanding of peripheral and central disease mechanisms.

## Conclusion

In summary, an integrated blood-based multi-omics approach identified convergent molecular signatures associated with RTT disease state and clinical severity, including alterations in translational, mitochondrial, chromatin-regulatory, cytoskeletal, immune, and metabolic processes. The alignment of these findings with pathways previously implicated in RTT supports their validity and relevance to the disorder, while their convergence across multiple molecular layers strengthens their biological significance and provides confidence in their robustness and potential utility as peripheral biomarkers. The present study was primarily directed towards biomarker discovery rather than characterisation of RTT biology. Therefore, it should be interpreted in terms of its initial contribution to the identification of accessible molecular signatures that can serve as biomarkers of diagnosis, patient classification, or therapeutic response. Validation in larger, independent cohorts will be required to refine these candidate biomarkers and to also determine whether distinct profiles are associated with specific *MECP2* variants, phenotypic profiles, or patterns of disease progression.

These findings establish a framework for objective biomarker discovery in RTT and provide an important step towards developing biologically informed measures capable of complementing diagnostic measures, supporting patient subtyping, and monitoring responses to available and novel treatments.

Integrated omics analysis offers substantial advantages to inform biomarker discovery by providing a comprehensive view of a disorder’s pathophysiology through the integration of transcriptomics, proteomics and metabolomics data. This systems-level approach improves the robustness of candidate biomarkers by cross-validating findings across molecular layers, reducing false positives and increasing clinical relevance. While individual omics data can be powerful, integrated omics analysis enables deeper mechanistic insights by linking biomarkers to disease pathways rather than simple associations (e.g. gene expression), accounting for individual heterogeneity, and supporting molecular subtyping. Significantly, using an integrated omics approach to identify biomarkers in patients’ blood enhances the predictive power to understand disease state and treatment response, increasing the likelihood of identifying accessible peripheral biomarkers that reflect the pathology in less accessible tissues such as the brain. This study also supports the use of omics analytical and statistical techniques to better evaluate and understand the complex biological processes underpinning RTT pathophysiology. This holistic approach may also prove useful for investigating biomarkers for other neurodevelopmental disorders and for providing comprehensive mechanistic insights.

While these data do not represent molecular changes in the brain, this study aimed to rather identify molecular changes in the circulating blood, using a minimally invasive, widely accessible method of monitoring that provides a more practical, scalable, and reproducible approach for both clinical diagnosis and management and therapeutic trials. These findings lay the groundwork and pave the way for future research where further studies are needed to confirm these peripheral molecular signatures As a whole, this study demonstrates that our identified latent factors may be potential biomarkers for disease state and response to interventions.

## Supplementary Information

Below is the link to the electronic supplementary material.

ESM 1 (XLSX 13.2 KB)

ESM 2 (XLSX 35.8 MB)

## Data Availability

The datasets generated during and/or analysed during the current study are available in the repository (access code 9667): https://repository.jpostdb.org/preview/192021941269a0cda77fb5e.

## References

[1] Rett A (1966) On a unusual brain atrophy syndrome in hyperammonemia in childhood. Wien Med Wochenschr 116(37):723–726 5300597

[2] Amir RE et al (1999) Rett syndrome is caused by mutations in X-linked MECP2, encoding methyl-CpG-binding protein 2. Nat Genet 23(2):185–188 10.1038/13810 10508514

[3] Cuddapah VA et al (2014) Methyl-CpG-binding protein 2 (MECP2) mutation type is associated with disease severity in Rett syndrome. J Med Genet 51(3):152–158 10.1136/jmedgenet-2013-102113 PMC4403764 24399845

[4] Keam SJ (2023) Trofinetide: first approval. Drugs 83(9):819–824 10.1007/s40265-023-01883-8 37191913

[5] Cope HL et al (2025) Feasibility and clinical utility of expanded genomic newborn screening in the Early Check program. Nat Med 31(11):3762–3771 10.1038/s41591-025-03945-8 PMC12520013 40913169

[6] Saby JN et al (2021) Multisite study of evoked potentials in Rett syndrome. Ann Neurol 89(4):790–802 10.1002/ana.26029 PMC8882338 33480039

[7] Downs J et al (2024) International workshop: what is needed to ensure outcome measures for Rett syndrome are fit-for-purpose for clinical trials? June 7, 2023, Nashville, USA. Trials 25(1):845 10.1186/s13063-024-08678-6 PMC11663341 39709426

[8] Kaufmann WE (2026) Rett syndrome in the therapeutic era: rethinking diagnosis, boundaries, clinical outcomes, and financing. Dev Med Child Neurol. 10.1111/dmcn.70516 42698224

[9] Zetterberg H, Bendlin BB (2026) Biofluid biomarkers in Alzheimer’s disease and other neurodegenerative dementias. Nature 650(8100):49–59 10.1038/s41586-025-10018-w 41639575

[10] Samaco RC et al (2009) Loss of MeCP2 in aminergic neurons causes cell-autonomous defects in neurotransmitter synthesis and specific behavioral abnormalities. Proc Natl Acad Sci U S A 106(51):21966–21971 10.1073/pnas.0912257106 PMC2799790 20007372

[11] Shulyakova N et al (2017) Mitochondrial dysfunction in the pathogenesis of Rett syndrome: implications for mitochondria-targeted therapies. Front Cell Neurosci 11:58 10.3389/fncel.2017.00058 PMC5348512 28352216

[12] Müller M (2019) Disturbed redox homeostasis and oxidative stress: potential players in the developmental regression in Rett syndrome. Neurosci Biobehav Rev 98:154–163 10.1016/j.neubiorev.2018.12.009 30639673

[13] Ehrhart F et al (2020) Integrated analysis of human transcriptome data for Rett syndrome finds a network of involved genes. World J Biol Psychiatry 21(10):712–725 10.1080/15622975.2019.1593501 30907210

[14] Neul JL et al (2020) Metabolic signatures differentiate Rett syndrome from unaffected siblings. Front Integr Neurosci 14:7 10.3389/fnint.2020.00007 PMC7052375 32161522

[15] Pascual-Alonso A et al (2023) Identification of molecular signatures and pathways involved in Rett syndrome using a multi-omics approach. Hum Genomics 17(1):85 10.1186/s40246-023-00532-1 PMC10503149 37710353

[16] Pecorelli A et al (2013) Genes related to mitochondrial functions, protein degradation, and chromatin folding are differentially expressed in lymphomonocytes of Rett syndrome patients. Mediators Inflamm 2013:137629 10.1155/2013/137629 PMC3876710 24453408

[17] Shovlin S, Tropea D (2018) Transcriptome level analysis in Rett syndrome using human samples from different tissues. Orphanet J Rare Dis 13(1):113 10.1186/s13023-018-0857-8 PMC6042368 29996871

[18] Zlatic SA et al (2022) Convergent cerebrospinal fluid proteomes and metabolic ontologies in humans and animal models of Rett syndrome. iScience 25(9):104966 10.1016/j.isci.2022.104966 PMC9437849 36060065

[19] Hagiwara S et al (2024) Comprehensive high-depth proteomic analysis of plasma extracellular vesicles containing preparations in Rett syndrome. Biomedicines. 10.3390/biomedicines12102172 PMC11504846 39457485

[20] Cicaloni V et al (2020) A proteomics approach to further highlight the altered inflammatory condition in Rett syndrome. Arch Biochem Biophys 696:108660 10.1016/j.abb.2020.108660 33159892

[21] Shahbazian MD et al (2002) Insight into Rett syndrome: MeCP2 levels display tissue- and cell-specific differences and correlate with neuronal maturation. Hum Mol Genet 11(2):115–124 10.1093/hmg/11.2.115 11809720

[22] Argelaguet R et al (2018) Multi-omics factor analysis-a framework for unsupervised integration of multi-omics data sets. Mol Syst Biol 14(6):e8124 10.15252/msb.20178124 PMC6010767 29925568

[23] Neul JL et al (2010) Rett syndrome: revised diagnostic criteria and nomenclature. Ann Neurol 68(6):944–950 10.1002/ana.22124 PMC3058521 21154482

[24] Percy AK et al (2025) Symptom onset in classic Rett syndrome: analysis of initial clinical severity scale entries. Ann Child Neurol Soc 3(3):152–157 10.1002/cns3.70017 PMC12380397 40880843

[25] Dobin A et al (2013) STAR: ultrafast universal RNA-seq aligner. Bioinformatics 29(1):15–21 10.1093/bioinformatics/bts635 PMC3530905 23104886

[26] Law CW et al (2014) Voom: precision weights unlock linear model analysis tools for RNA-seq read counts. Genome Biol 15(2):R29 10.1186/gb-2014-15-2-r29 PMC4053721 24485249

[27] Ritchie ME et al (2015) Limma powers differential expression analyses for RNA-sequencing and microarray studies. Nucleic Acids Res 43(7):e47 10.1093/nar/gkv007 PMC4402510 25605792

[28] Robinson MD, McCarthy DJ, Smyth GK (2010) EdgeR: a Bioconductor package for differential expression analysis of digital gene expression data. Bioinformatics 26(1):139–140 10.1093/bioinformatics/btp616 PMC2796818 19910308

[29] Risso D et al (2014) Normalization of RNA-seq data using factor analysis of control genes or samples. Nat Biotechnol 32(9):896–902 10.1038/nbt.2931 PMC4404308 25150836

[30] Engholm-Keller K, Larsen MR (2016) Improving the phosphoproteome coverage for limited sample amounts using TiO_2_-SIMAC-HILIC (TiSH) phosphopeptide enrichment and fractionation. Phospho-proteomics: methods and protocols. pp 161–177 10.1007/978-1-4939-3049-4_11 26584925

[31] Engholm-Keller K et al (2019) The temporal profile of activity-dependent presynaptic phospho-signalling reveals long-lasting patterns of poststimulus regulation. PLoS Biol 17(3):e3000170 10.1371/journal.pbio.3000170 PMC6415872 30822303

[32] Tsang E et al (2024) Ketogenic diet modifies ribosomal protein dysregulation in KMT2D Kabuki syndrome. EBioMedicine 104:105156 10.1016/j.ebiom.2024.105156 PMC11134553 38768529

[33] Wu T et al (2021) clusterProfiler 4.0: a universal enrichment tool for interpreting omics data. Innovation. 10.1016/j.xinn.2021.100141 PMC8454663 34557778

[34] Supek F et al (2011) REVIGO summarizes and visualizes long lists of gene ontology terms. PLoS ONE 6(7):e21800 10.1371/journal.pone.0021800 PMC3138752 21789182

[35] Storey JD (2002) A direct approach to false discovery rates. J R Stat Soc Ser B Stat Methodol 64(3):479–498

[36] Ford L et al (2020) Precision of a clinical metabolomics profiling platform for use in the identification of inborn errors of metabolism. J Appl Lab Med 5(2):342–356 10.1093/jalm/jfz026 32445384

[37] Miller MJ et al (2015) Untargeted metabolomic analysis for the clinical screening of inborn errors of metabolism. J Inherit Metab Dis 38:1029–1039 10.1007/s10545-015-9843-7 PMC4626538 25875217

[38] Molania R et al (2019) A new normalization for NanoString nCounter gene expression data. Nucleic Acids Res 47(12):6073–6083 10.1093/nar/gkz433 PMC6614807 31114909

[39] Stekhoven DJ, Bühlmann P (2012) MissForest—non-parametric missing value imputation for mixed-type data. Bioinformatics 28(1):112–118 10.1093/bioinformatics/btr597 22039212

[40] Subramanian I et al (2020) Multi-omics data integration, interpretation, and its application. Bioinform Biol Insights. 10.1177/1177932219899051 PMC7003173 32076369

[41] Ricciardi S et al (2011) Reduced AKT/mTOR signaling and protein synthesis dysregulation in a Rett syndrome animal model. Hum Mol Genet 20(6):1182–1196 10.1093/hmg/ddq563 21212100

[42] Rodrigues DC et al (2020) Shifts in ribosome engagement impact key gene sets in neurodevelopment and ubiquitination in Rett syndrome. Cell Rep 30(12):4179-4196.e11 10.1016/j.celrep.2020.02.107 32209477

[43] Bengtson MH, Joazeiro CA (2010) Role of a ribosome-associated E3 ubiquitin ligase in protein quality control. Nature 467(7314):470–473 10.1038/nature09371 PMC2988496 20835226

[44] Tannahill GM et al (2013) Succinate is an inflammatory signal that induces IL-1β through HIF-1α. Nature 496(7444):238–242 10.1038/nature11986 PMC4031686 23535595

[45] Nan X et al (1998) Transcriptional repression by the methyl-CpG-binding protein MeCP2 involves a histone deacetylase complex. Nature 393(6683):386–389 10.1038/30764 9620804

[46] Lyst MJ, Bird A (2015) Rett syndrome: a complex disorder with simple roots. Nat Rev Genet 16(5):261–275 10.1038/nrg3897 25732612

[47] Lilja T et al (2013) Novel alterations in the epigenetic signature of MeCP2-targeted promoters in lymphocytes of Rett syndrome patients. Epigenetics 8(3):246–251 10.4161/epi.23752 PMC3669117 23348913

[48] Watson JA et al (2026) MeCP2 requires interactions with nucleosome linker DNA to read chromatin DNA methylation. Nat Commun 17(1):5374 10.1038/s41467-026-71741-0 PMC13276077 41997957

[49] Kaufmann WE, MacDonald SM, Altamura CR (2000) Dendritic cytoskeletal protein expression in mental retardation: an immunohistochemical study of the neocortex in Rett syndrome. Cereb Cortex 10(10):992–1004 10.1093/cercor/10.10.992 11007550

[50] Varderidou-Minasian S et al (2020) Quantitative proteomic analysis of Rett iPSC-derived neuronal progenitors. Mol Autism 11(1):38 10.1186/s13229-020-00344-3 PMC7251722 32460858

[51] Lux SE (2016) Anatomy of the red cell membrane skeleton: unanswered questions. Blood 127(2):187–199 10.1182/blood-2014-12-512772 26537302

[52] Rana AP et al (1993) Cloning of human erythroid dematin reveals another member of the villin family. Proc Natl Acad Sci U S A 90(14):6651–6655 10.1073/pnas.90.14.6651 PMC46990 8341682

[53] Voskoboinik I, Whisstock JC, Trapani JA (2015) Perforin and granzymes: function, dysfunction and human pathology. Nat Rev Immunol 15(6):388–400 10.1038/nri3839 25998963

[54] Elgueta R et al (2009) Molecular mechanism and function of CD40/CD40L engagement in the immune system. Immunol Rev 229(1):152–172 10.1111/j.1600-065X.2009.00782.x PMC3826168 19426221

[55] Takeda A et al (1994) Molecular cloning of the CD45-associated 30-kDa protein. J Biol Chem 269(4):2357–2360 8300558

[56] O’Driscoll CM, Kaufmann WE, Bressler JP (2013) MeCP2 deficiency enhances glutamate release through NF-κB signaling in myeloid derived cells. J Neuroimmunol 265(1–2):61–67 10.1016/j.jneuroim.2013.09.002 24268627

[57] Kishi N et al (2016) Reduction of aberrant NF-κB signalling ameliorates Rett syndrome phenotypes in Mecp2-null mice. Nat Commun 7:10520 10.1038/ncomms10520 PMC4740176 26821816

[58] Cappuccio G et al (2019) Sphingolipid metabolism perturbations in Rett syndrome. Metabolites. 10.3390/metabo9100221 PMC6835521 31658741

[59] Zandl-Lang M et al (2022) Changes in the cerebrospinal fluid and plasma lipidome in patients with Rett syndrome. Metabolites. 10.3390/metabo12040291 PMC9026385 35448478

[60] Segatto M et al (2014) Cholesterol metabolism is altered in Rett syndrome: a study on plasma and primary cultured fibroblasts derived from patients. PLoS ONE 9(8):e104834 10.1371/journal.pone.0104834 PMC4130597 25118178

[61] Hamberger A et al (1992) Elevated CSF glutamate in Rett syndrome. Neuropediatrics 23(4):212–213 10.1055/s-2008-1071344 1357572

[62] Lappalainen R, Riikonen RS (1996) High levels of cerebrospinal fluid glutamate in Rett syndrome. Pediatr Neurol 15(3):213–216 10.1016/s0887-8994(96)00218-4 8916158

[63] O’Driscoll CM et al (2015) Methyl CpG binding protein 2 deficiency enhances expression of inflammatory cytokines by sustaining NF-κB signaling in myeloid derived cells. J Neuroimmunol 283:23–29 10.1016/j.jneuroim.2015.04.005 26004152

